# “Smart” Materials Based on Cellulose: A Review of the Preparations, Properties, and Applications

**DOI:** 10.3390/ma6030738

**Published:** 2013-02-28

**Authors:** Xiaoyun Qiu, Shuwen Hu

**Affiliations:** 1 Department of Environmental Sciences & Engineering, College of Resources & Environmental Sciences, China Agricultural University, Beijing 100193, China; E-Mail: qiuxiaoyun@live.cn; 2 Department of Environmental Sciences & Engineering, College of Resources & Environmental Sciences, China Agricultural University, Beijing 100193, China

**Keywords:** cellulose, stimuli-responsive, smart materials, drug delivery

## Abstract

Cellulose is the most abundant biomass material in nature, and possesses some promising properties, such as mechanical robustness, hydrophilicity, biocompatibility, and biodegradability. Thus, cellulose has been widely applied in many fields. “Smart” materials based on cellulose have great advantages—especially their intelligent behaviors in reaction to environmental stimuli—and they can be applied to many circumstances, especially as biomaterials. This review aims to present the developments of “smart” materials based on cellulose in the last decade, including the preparations, properties, and applications of these materials. The preparations of “smart” materials based on cellulose by chemical modifications and physical incorporating/blending were reviewed. The responsiveness to pH, temperature, light, electricity, magnetic fields, and mechanical forces, *etc.* of these “smart” materials in their different forms such as copolymers, nanoparticles, gels, and membranes were also reviewed, and the applications as drug delivery systems, hydrogels, electronic active papers, sensors, shape memory materials and smart membranes, *etc.* were also described in this review.

## 1. Introduction

**Figure 1:**
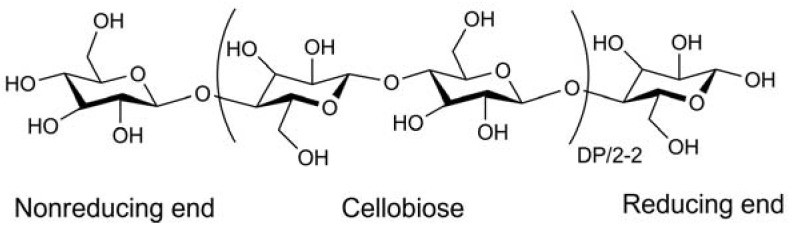
Molecular structure of cellulose.

Cellulose is the most abundant renewable organic material produced in the biosphere, with approximately 5 × 10^11^ metric tons being generated yearly. Unfortunately, a mere 2% is recovered industrially [1]. Cellulose is a linear syndiotactic homopolymer composed of *D*-anhydroglucopyranose units (AGUs), which are linked by *β*-(1→4)-glycosidic bonds (Figure 1). Due to the high intensity of hydroxyl groups along the skeleton, the extended network of hydrogen bonds (intra- and inter-molecular bonds) are formed. Consequently, two structure regions can be found: the crystalline region and the amorphous region [2].

Cellulose is a colorless, odorless, and nontoxic solid polymer, and possesses some promising properties, such as great mechanical strength, biocompatibility, hydrophilicity, relative thermostabilization, high sorption capacity, and alterable optical appearance [2]. These properties enable cellulose to be applied to a vast array of fields. Some critical reviews concerning applications of materials based on cellulose in typical forms are listed in Table 1.

**Table 1:** Applications of cellulose in different forms illustrated in reviews.

Material forms	Applications	References
fiber	fiber, reinforcement material, biomaterial, magnetic paper *et al.*	[3,4,5,6,7]
film/membrane	drug delivery, separation, water treatment, package, optical media, biomembrane, adsorption, *etc.*	[3,4,5,6,7]
nanocomposite	biomaterials, drug delivery, reinforcement material, barrier film, membrane, conductive material, adhesion, *etc.*	[14,15,16,17,18,19,20]
npolymer	polymer drug delivery, biomaterial, water treatment, thickener, stabilizer, *etc.*	[14,15,16,17,18,19,20]

**Figure 2:**
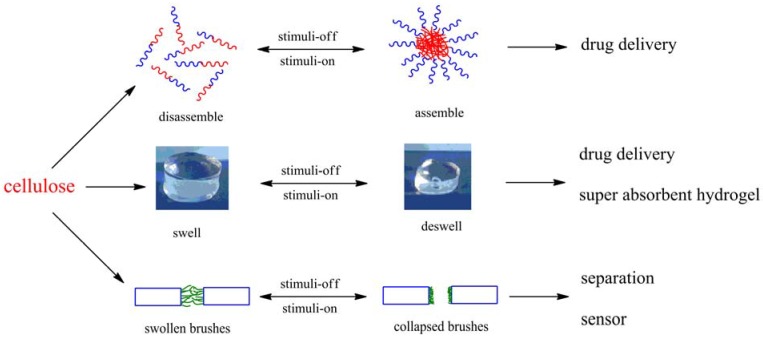
Examples of “smart” materials based on cellulose and their possible applications.

Despite those specific descriptions regarding applications of materials based on cellulose in the above reviews, cellulose can be used to fabricate “smart” materials, which present intelligent behaviors under environmental stimulus. “Smart” material is defined as one in which a key material property could be altered in a controlled manner in response to the introduction of a predetermined external stimulus [25]. These stimuli-responsive materials might be utilized to undergo such changes as specimen shape, mechanical rigidity/flexibility, opacity, and porosity. Due to the intriguing property changes, “smart” materials have great potential in many applications, especially as biomaterials and drug carriers; some examples of material forms and their applications are given in Figure 2. Amphiphilic polymers can assemble/disassemble in water under certain stimulus changes, and drug-loaded micelles can be used as drug delivery systems. Hydrogels undergo swelling and deswelling in response to environmental changes and thus can also be applied for drug delivery, and for super absorbent hydrogels. And stimuli-responsive polymer-grafted membranes can regulate their pore sizes through polymer swelling and shrinking in response to stimulus. This kind of membrane can be fabricated to be separation membranes and sensors. The stimuli on/off switch can be produced by changes of pH, temperature, ionic concentration, *etc.* “Smart” materials based on cellulose inherit its unique properties, such as strong mechanical strength, and biocompatibility, thus studies on “smart” materials based on cellulose have bloomed during the last decade.

However, to our knowledge, there are very few reviews on “smart” materials based on cellulose, and only hydrogels were referred to [26,27,28]. Herein, we aim to review the preparations, properties, and applications of “smart” materials based on cellulose developed in the last decade, including smart hydrogels prepared with cellulose. We will introduce the fabrication strategies of “smart” materials using cellulose as the main materials or additives, and focus on the properties of these materials and their potential applications.

## 2. Preparation Strategies

**Figure 3:**
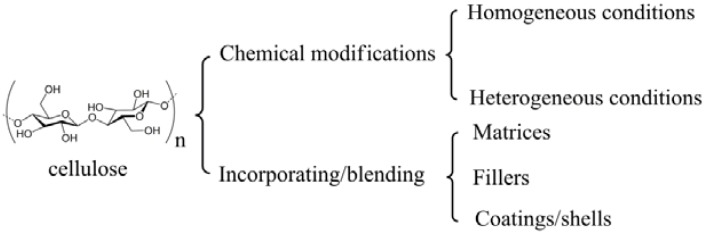
Preparation strategies of “smart” materials based on cellulose.

“Smart” materials based on cellulose can be fabricated through chemical modifications or physical incorporating/blending (Figure 3). Chemical modifications can be conducted both in homogeneous conditions and in heterogeneous conditions. In the processes of incorporating/blending, cellulose or cellulose derivatives act as matrices, fillers, or coatings/shells. The prepared “smart” materials based on cellulose are usually in the forms of copolymers, aggregates, particles, gels, fibers, membranes, and films.

### 2.1. Chemical Modifications

Cellulose has three alcoholic hydroxyl groups in each of its AGUs, and chemical modifications can be exclusively performed on these hydroxyls with practical relevance. The primary hydroxyl group at C-6 and the two secondary ones at C-2 and C-3 can participate in all the classical reactions as the alcoholic hydroxyl group does, including esterification, etherification, and oxidation reactions. Chemical modifications can be conducted both in heterogeneous and homogeneous conditions. Due to the high crystallinity, cellulose can be only dissolved in limited solvents, so many modifications are conducted in heterogeneous conditions. Since chemical reactions occur only at the surface layer in heterogeneous conditions, the gross structure of the cellulose sample can be largely maintained. In homogeneous conditions, the original supermolecular structure of the sample is destroyed and the limitation of the completeness of the chemical reaction can be minimized, thus well-defined cellulose materials can be obtained through chemical modifications in homogeneous conditions.

#### 2.1.1. Modifications in Homogeneous Conditions

Homogeneous conditions can be realized by dissolving cellulose in nonderivatizing solvents, e.g., *N*,*N*-dimethylacetamide/LiCl, and in derivatizing solvents, e.g., N_2_O_4_/DMF, or by dissolution of cellulose derivatives in suitable solvents, employing the substituents as protecting groups, leaving groups, or starting groups for consecutive reactions. Since solvents for cellulose are either toxic, such as N_2_O_4_/DMF, or hard to remove, such as *N*,*N*-dimethylacetamide/LiCl, most of the chemical modifications in homogeneous conditions start with cellulose derivatives, which can be dissolved in water or common organic solvents, including carboxymethyl cellulose (CMC), hydroxypropyl cellulose (HPC), hydroxyethyl cellulose (HEC), and cellulose acetate (CA). More interestingly, CMC with a high degree of substitution (DS) of carboxymethyl groups is a pH-responsive polyelectrolyte similar to poly(acrylic acid) [29,30], with p*K*_a_ around 3–4 [31]. HPC is a temperature-responsive derivative of natural macromolecules, and exhibits a lower critical solution temperature (LCST) in aqueous solution at about 41 °C [32,33] and a remarkable hydration–dehydration change in aqueous solution in response to relative changes in temperature around the LCST. By combining with other stimulus-responsive polymers, these cellulose derivatives demonstrate a variety of intelligent behaviors.

**Figure 4:**
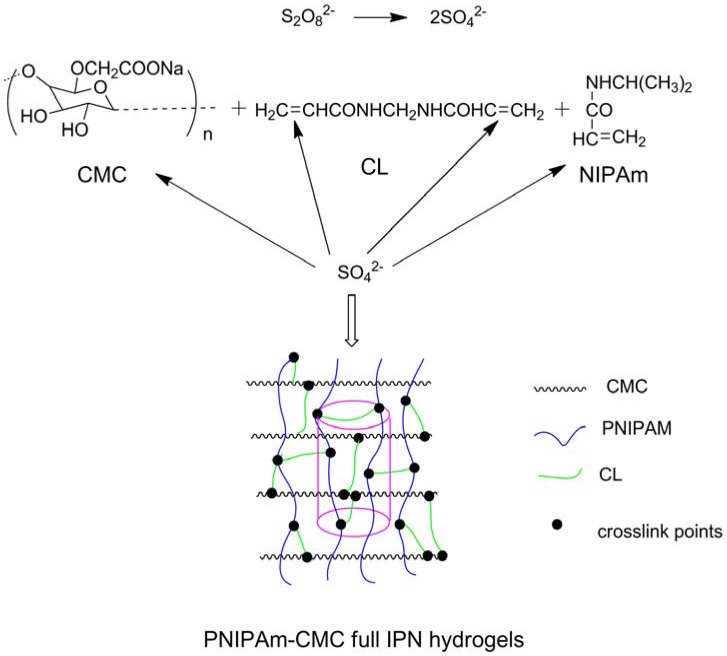
Cylindrical-shaped PNIPAm-CMC full interpenetrating networks (IPN) hydrogels were prepared by the simultaneous radical crosslinking of CMC and PNIPAm polymer chains. The CMC/PNIPAm weight ratio was 5.0/95.0 and the hydrogels were prepared at 18 °C. PNIPAm: Poly(*N*-isopropylacrylamide); CMC: carboxymethyl cellulose. Reprinted with permission from [30]. Copyright 2010 Springer.

CMC has been widely applied in many fields, especially in drug delivery systems. Many researchers have exploited the combination of polyacids with CMC to fabricate responsive polymers or hydrogels. Weak polyacids (or polybases), which undergo an ionization/deionization transition from pH 4–8, are utilized as pH-responsive polymers. And those bearing the carboxylic group with p*K*_a_ around 5–6 are the most representative weak polyacids, such as poly(acrylic acid) (PAA) and poly(methacrylic acid) (PMAA) [34]. Pal *et al.* prepared a pH-sensitive hydrogel membrane by esterification of sodium CMC dissolved in water with acryloyl chloride added dropwise in methyl ethyl ketone [35]. Another method often used is free-radical polymerization. Dissolved CMC was added with acrylic acid (AA) neutralized by NaOH solution, the crosslinker *N*,*N*′-methylene-*bis*-acrylamide (MBA), the initiator ammonium persulfate (APS), and the modifier rectorite micropowder. The polymerization was conducted under nitrogen for 3 h at 70 °C to obtain superabsorbent nanocomposites responsive to various saline, pH, and hydrophilic organic solvent/water solutions [36]. CMC grafted with polyacrylamide and polyacrylonitrile were synthesized similarly, and observed with pH-responsive features [37,38]. Poly(*N*-isopropylacrylamide) (PNIPAm) and random copolymers of ethylene oxide and propylene oxide (PEPO) are very good candidates with LCST around body temperature [39,40] for studies focusing on biomaterials. PNIPAm-CMC interpenetrating polymeric networks were prepared by crosslinking with *N*,*N*,*N*′,*N*′-tetramethylethylene diamine *via* free-radical polymerization initiated by potassium persulfate (KPS) (Figure 4) and showed temperature- and pH-responsive swelling behaviors [30]. Free-radical polymerization initiated by KPS was also applied by Cha *et al.* to fabricate CMC/PNIPAm hydrogels in water using carboxylated nanocrystalline cellulose [41]. An amino-terminated PEPO was grafted onto the CMC skeleton by esterification of amino groups and carboxyl groups in acidic aqueous solution, and the obtained thickeners were responsive to temperature [39].

**Figure 5:**
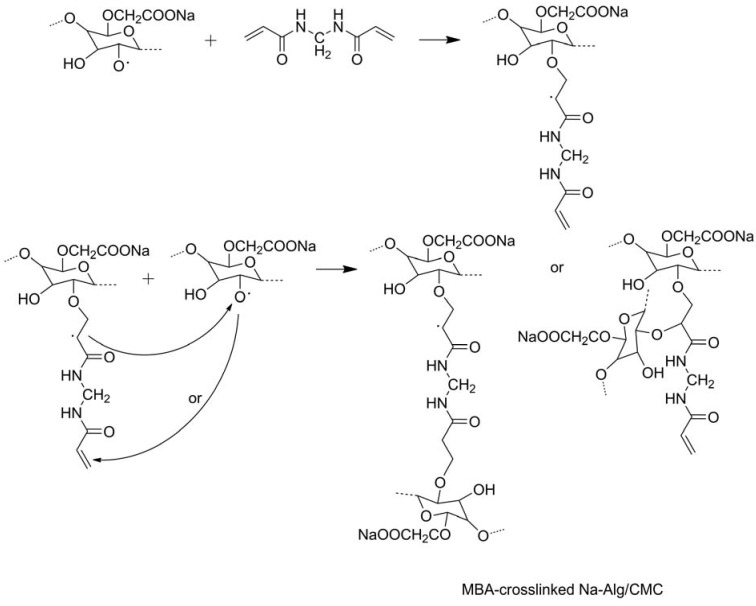
General mechanism for radical crosslinking of Na-Alg/CMC mixture in the presence of *N*,*N*′-methylene-*bis*-acrylamide (MBA). Reprinted with permission from [46]. Copyright 2006 Elsevier.

Crosslinking of CMC is another method to regulate responsive swelling properties of the hydrogels. CMC, along with HEC, was dissolved in water before citric acid was added as a crosslinker to form slightly crosslinked hydrogels, which showed temperature-responsive swelling ability [42]. Divinylsulfone (DVS), as a crosslinker for the CMC and HEC mixture, was adopted to form ionic and pH-sensitive hydrogels [43]. Quaternized cellulose (QC) that included the CMC solution was crosslinked with epichlorohydrin to fabricate pH- and salt-responsive hydrogels [44]. Chang *et al.* prepared saline-responsive hydrogels by dissolving cellulose and CMC in NaOH/urea aqueous system and crosslinking cellulose and CMC with epichlorohydrin [45]. Water-soluble sodium alginate (Na-Alg) was mixed with CMC and crosslinked by MBA via free-radical polymerization to obtain saline- and pH-responsive hydrogels (Figure 5) [46], and Alg-CMC bi-polymer hydrogels crosslinked with ferric and calcium ions were achieved by a microfluidic approach and *in situ* encapsulation of BSA by versatile control over the local fluids [47]. Montmorillonite was mixed with CMC in aqueous solution and MBA was added in the solution as a crosslinker, and the reaction was conducted with electron beam irradiation [48]. The prepared hydrogels were sensitive to inorganic salts aqueous solution, physical saline water, and synthetic urine, showing smart swelling and shrinking behaviors.

**Figure 6:**
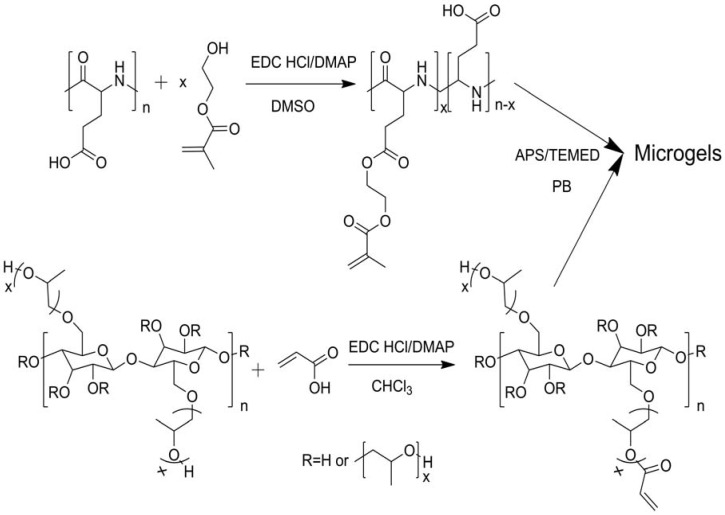
Synthesis route of microgels prepared from hydroxypropyl cellulose (HPC) [33].

**Figure 7:**
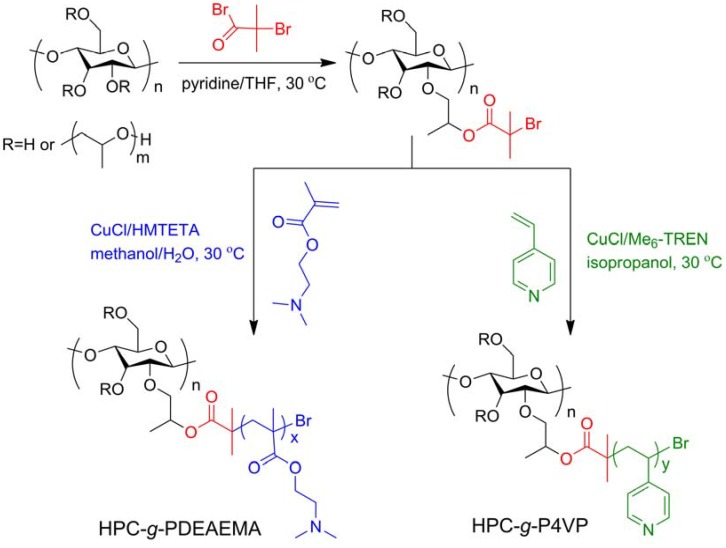
Synthesis route of the poly(*N*,*N*-dimethyl aminoethyl methacrylate) (PDMAEMA) and poly(4-vinyl pyridine) (P4VP) grafted HPC via atom transfer radical polymerization (ATRP) [53,54].

**Figure 8:**
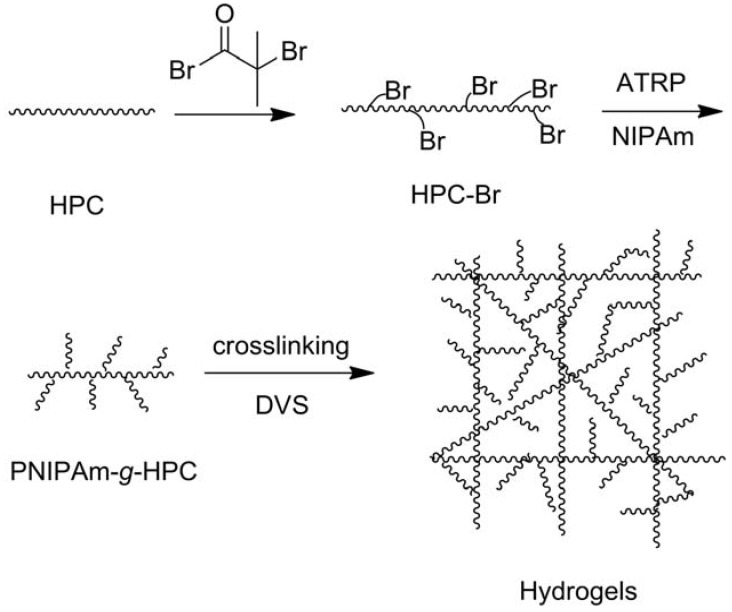
Schematic diagram illustrating the processes for the preparation of the PNIPAm-*g*-HPC copolymers via ATRP of NIPAm from the alkyl bromide-functionalized HPC macroinitiator and the formation of stimuli-responsive hydrogels via crosslinking. Reprinted with permission from [55]. Copyright 2010 American Chemical Society.

**Figure 9:**
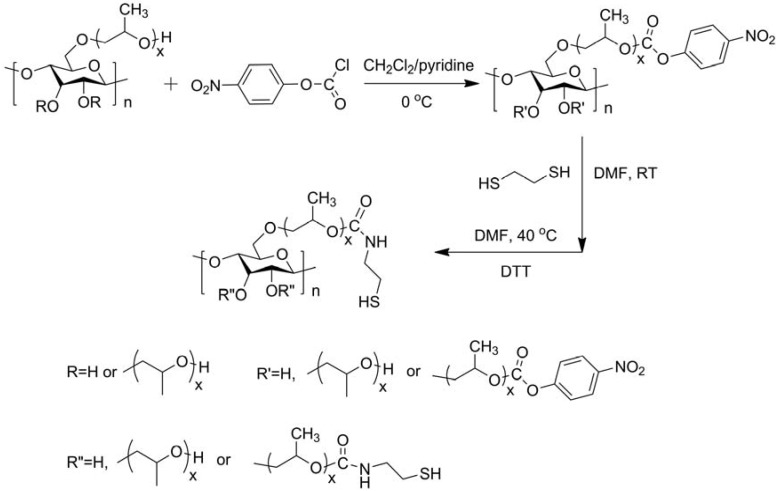
Synthesis route of the thiolated HPC derivatives. Reprinted with permission from [58]. Copyright 2010 Royal Society of Chemistry.

“Smart” materials based on HPC are also intensively studied these years because of its solubility in water and temperature-responsive property, and the fabrication strategies are similar to those made with CMC. Polyacids were grafted onto or interpenetrated with HPC by free-radical polymerization in water initiated by oxidant [49,50,51], and the prepared hydrogels were temperature-responsive, or temperature- and pH-responsive. Chen’s research group developed a two-step method to fabricate thermo- and pH-responsive hydrogels based on HPC. HPC was firstly grafted with AA by esterification, then PAA or poly(L-glutamic acid–hydroxyethyl methacrylate) was grafted from HPC through free-radical polymerization initiated by APS (Figure 6) [32,33]. HPC-based interpenetrating networks with polyacrylamide were synthesized in aqueous medium by simultaneous γ-rays initiation in the presence of MBA by Chauhan and Mahajan [52]. HPC was firstly esterified by 2-bromoisobutyryl bromide (BriB), and then Poly(*N*,*N*-dimethyl aminoethyl methacrylate) (PDMAEMA) [53] and poly(4-vinyl pyridine) (P4VP) [54] were grafted from HPC backbones via atom transfer radical polymerization (ATRP) in homogeneous conditions (Figure 7), and the polymers were both thermo- and pH-responsive. PNIPAm was grafted from HPC similarly, and the obtained polymers were further modified by crosslinking with DVS (Figure 8) [55]. The prepared hydrogel was thermo-responsive. Interpenetrating networks of HPC and PNIPAm [56] and poly[(*N*-*tert*-butylacrylamide)-*co*-acrylamide] [57] with temperature-responsive properties were prepared by free-radical polymerization initiated by APS. Tan *et al.* reported a thermo- and redox-sensitive nanogel fabricated by self-association of thiolated HPC (HPC-SH) (Figure 9) [58]. HPC was first activated by 4-nitrophenyl chloroformate dissolving in CH_2_Cl_2_ with pyridine as a catalyst. The modified HPC was converted to HPC-SH in the presence of cysteamine. Then the HPC-SH was dissolved in DMF with DL-dithiothreitol and then was dialyzed against water to get the HPC-SH aqueous solution. Disulfide bonds were formed to re-crosslink the collapsed HPC chains into nanogels while the HPC-SH solution was cured at 45 °C (above the LCST of HPC) with dimethyl sulfoxide (DMSO) as an oxidant.

**Figure 10:**
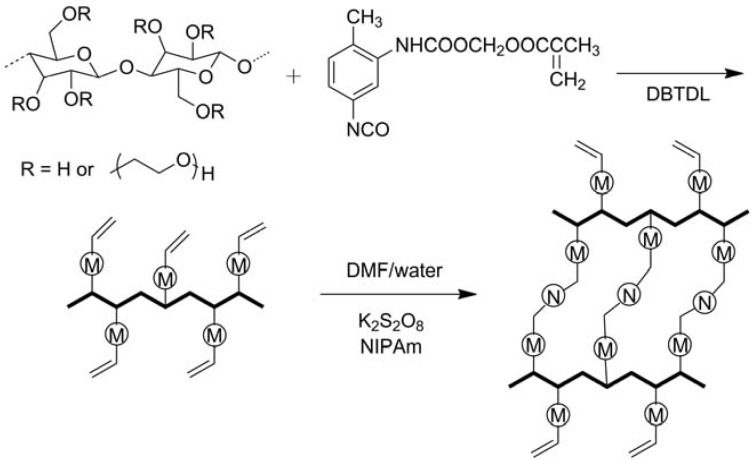
Synthesis route of temperature-responsive hydrogels based on HEC. Reprinted with permission from [60]. Copyright 2010 Taylor & Francis.

Other cellulose derivatives were also utilized to synthesize “smart” materials. Binary graft copolymers HEC-*g*-PNIPAm-PAA were synthesized through successive cerium(IV)-initiated free-radical copolymerization of NIPAm and AA from HEC backbone in water [59]. Peng and Chen reported another way to prepare temperature-sensitive hydrogels based on HEC (Figure 10) [60]. Monoblocked diisocyanate was firstly synthesized by reaction of 2,4-toluene diisocyanate, 1,4-dioxane, and 2-hydroxyethyl methacrylate. The prepared monoblocked diisocyanate bearing isocyano groups of 2,4-toluene diisocyanate and alkene groups of 2-hydroxyethyl methacrylate was grafted onto HEC backbone through reaction of isocyano groups with hydroxyl groups on HEC. Then the modified HEC with pendant alkene groups was crosslinked with NIPAm initiated by KPS in DMF/water system to obtain the temperature-sensitive hydrogels. EC was grafted with NIPAm in chloroform/DMSO solvent system initiated by APS, then the EC-*g*-PNIPAm was dissolved in CH_2_Cl_2_ with allopurinol as a model drug to produce thermo-responsive drug delivery microparticles using a B-191 mini spray-dryer [61]. Yuan *et al.* prepared a type of tunable pH- and temperature-responsive EC brush polymers with mono and dual side chains by click chemistry [62]. EC was firstly reacted with BriB to get 2-bromoisobutyryl EC (EC-Br) in anhydrous chloroform and azide-EC (EC-N_3_) was prepared by reaction of EC-Br with NaN_3_ in DMF. Click chemistry was carried out in DMF solution of mixtures of EC-N_3_ and alkynyl-PDMAEMA and/or alkynyl-poly(2-(2-methoxyethoxy)ethyl methacrylate)-*co*-oligo(ethylene glycol) methacrylate) [alkynyl-P(MEO_2_MA-*co*-OEGMA)] in the presence of CuBr. CA dissolved in glacial acetic acid was crosslinked with PAA and the pH-responsive membrane was produced by forming on a flat glass mold [63]. CA and poly[styrene-*co*-(maleic sodium anhydride)] were dissolved in DMAc/acetone mixed solvent and electrospun to form nanofibrous mats and were heat-treated to allow crosslinking reactions to take place [64]. The crosslinked hydrogel nanofibers supported on cellulose showed improved dimensional stability upon immersion in aqueous solutions and were pH-responsive.

**Figure 11:**
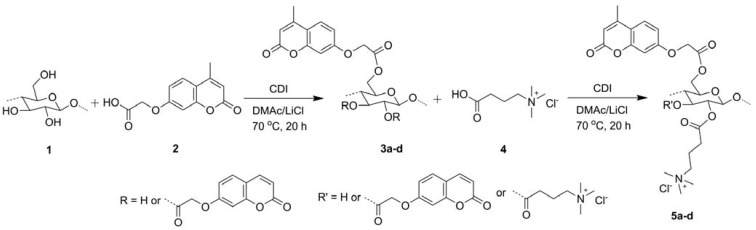
Synthesis route of cellulose 2-[(4-methyl-2-oxo-2H-chromen-7-yl) oxy] acetates (3a-d) and cellulose 2-[(4-methyl-2-oxo-2H-chromen-7-yl) oxy] acetate [4-(*N*,*N*,*N*-trimethylamonium) chloride] butyrates (5a-d) via *in situ* activation of 2-[(4-methyl-2-oxo-2H-chromen-7-yl) oxy] acetic acid (2) and (3-carboxypropyl) trimethylammonium chloride (4) with *N*,*N*-carbonyldiimidazole (CDI) in DMAc/LiCl. Reprinted with permission from [67]. Copyright 2012 Springer.

So far there has been little research on the direct homogeneous graft polymerization on cellulose backbone because of the poor solubility of cellulose and undesired chain degradation of the backbone in specific solvents [65]. Sui *et al.* utilized 1-allyl-3-methylimidazolium chloride and DMF as solvent system to synthesize cellulose macroinitiator by esterification with BriB, and the macroinitiator was grafted with PDMAEMA via ATRP to get pH-responsive cellulose-*g*-PDMAEMA copolymers [66]. Cellulose was esterified with photoactive and cationic carboxylic acids in DMAc/LiCl system by successive reaction with 2-[(4-methyl-2-oxo-2H-chromen-7-yl) oxy] acetic acid and (3-carboxypropyl) trimethylammonium chloride in the presence of *N*,*N*-carbonyldiimidazole (CDI), and the obtained cellulose derivative was water soluble and photoactive (Figure 11) [67].

Cai and Kim prepared an electroactive paper actuator made by cellulose/polyurethane semi-interpenetrating polymer networks [68]. Cotton cellulose was first dissolved in DMAc/LiCl solvent system and then mixed with polyurethane (PU) prepolymer made by reaction of hexamethylene diisocyanate and poly[di(ethylene glycol) adipate]. The cellulose/PU solution was added with 1,1,1-tris(hydroxymethyl) propane as crosslinker and was spin-coated on wafer and cured to get the final electroactive films.

#### 2.1.2. Modifications in Heterogeneous Conditions

Modification of cellulose in heterogeneous conditions usually performed in the forms of cellulose nanocrystals (CNCs), films/membranes, fibers, and cellulose particle suspensions. CNCs have gained much attention not only because of their unsurpassed quintessential physical and chemical properties, but also because of their inherent renewability and sustainability in addition to their abundance. They have been the subject of a wide array of research efforts as reinforcing agents in nanocomposites due to their low cost, availability, renewability, light weight, nanoscale dimension, and unique morphology [69]. The chemical modifications of cellulose in heterogeneous conditions are normally conducted after swelling in suitable solvents.

**Figure 12:**
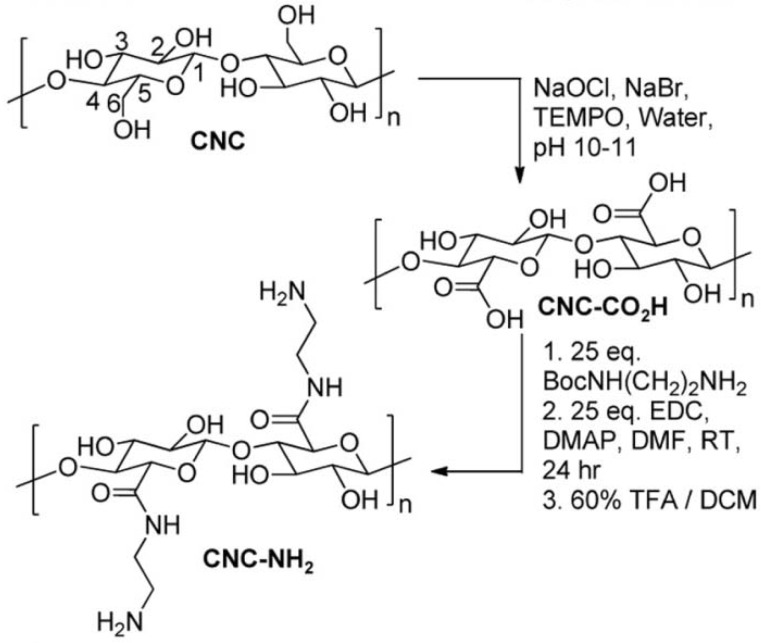
Synthesis route of pH-responsive CNCs (carboxylated CNCs and amine-functionalized CNCs). Reprinted with permission from [72]. Copyright 2012 American Chemical Society.

**Figure 13:**
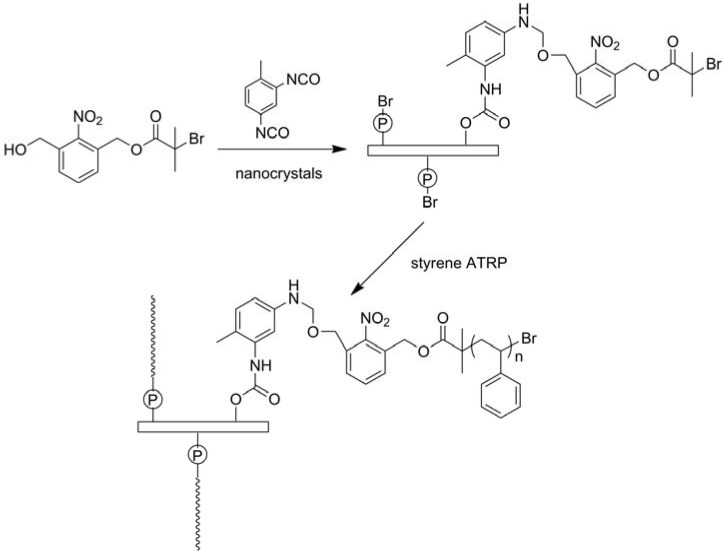
Synthesis route of nanoparticles with photocleavable PS grafts. Reprinted with permission from [73]. Copyright 2012 Royal Society of Chemistry.

The main process for the isolation of CNCs from cellulose fibers is based on acid hydrolysis [69,70,71]. Disordered or paracrystalline regions of cellulose are preferentially hydrolyzed, whereas crystalline regions having a higher resistance to acid attack remain intact. Followed with an acid treatment that hydrolyzes the cellulose, then cellulose rod-like nanocrystals are produced [69]. Zoppe *et al.* prepared temperature-responsive CNCs by grafting PNIPAm brushes from CNCs via surface-initiated single-electron transfer living radical polymerization [70]. Initiator-modified CNCs was prepared by reacting with BriB initiator in the tetrahydrofuran suspension, and the polymerization was conducted with CuBr and *N*,*N*,*N*′,*N*′′,*N*′′-pentamethyldiethylenetriamine. It was expected that the suspension stability, interfacial interactions, friction, and other properties of grafted CNCs can be controlled by temperature changes and offer a unique platform for further development of stimuli-responsive nanomaterials. Azzam *et al.* described the grafting of thermosensitive Jeffamine macromolecules on the surface of CNCs using a “grafting onto” strategy [71]. CNCs were firstly carboxylated by TEMPO oxidation, followed by grafting of amine-terminated Jeffamine through peptidic coupling. Way *et al.* reported a method to fabricate pH-responsive CNCs gels and nanocomposites, and the synthesis route is illustrated in Figure 12 [72]. Carboxylated CNCs was prepared by TEMPO oxidation, and amine-functionalized CNCs was prepared by further reaction of carboxylated CNCs with *tert*-butyl (2-aminoethyl) carbamate, and the Boc protecting groups was removed. The prepared carboxylated CNCs and amine-functionalized CNCs showed pH-responsive and composites of poly(vinyl acetate) (PVAc) filled with prepared CNCs also showed mechanical strength changes with pH variation. CNCs were grafted with photocleavable polymeric chains using ATRP (Figure 13) [73]. The nanoparticle synthesis was comprised of two steps: Grafting of a photosensitive moiety bearing an ATRP initiating site onto the surface of CNCs by linking by toluene diisocyanate, followed by surface initiated ATRP of polystyrene from the modified surface outward. The polystyrene brushes were sensitive to UV irradiation and could be degrafted from the surface of CNCs by UV irradiation.

Cellulose films/membranes are usually manufactured with cellulose esters because of their mechanical strength and ease of accessibility. Pan *et al.* prepared double stimuli-responsive RC membranes grafted with block copolymer of PAA and PNIPAm by ATRP method [74]. The RC membrane was first immobilized with ATRP initiator BriB, and PAA and PNIPAm were successively grafted with membranes suspended in water via ATRP. We reported that double stimuli-responsive membranes could be fabricated by simultaneously grafted PNIPAm and PDEAEMA onto modified cellulose membrane using a transdermal diffusion device via surface-initiated activators regenerated by electron transfer ATRP [75]. Cerium ammonium nitrate (CAN) was used to initiate radical polymerization to fabricate PNIPAm grafted CA ultrafiltration membranes by Gorey and Escobar [76]. NIPAm monomers or NIPAm prepolymers was dissolved in nitric acid and mixed with CAN and CA membranes and the polymerization was carried out in an Aldrich Atmosbag (Z530204-1EA). Photografting of MAA and NIPAm on RC film was carried out in water to prepare pH-responsive and temperature-responsive cellulose films [77]. Gorey *et al.* reported a method to build temperature-responsive CA membrane filters for microbial sensing, which was comprised of two steps: HPC was first crosslinked with DVS and then the crosslinked HPC was grafted onto a CA membrane linked by DVS [78].

Fiber is the most widely used form of cellulose since long before cellulose had been known by humankind and it will be the dominant application form in the future. Cellulose fiber was used as a support to incorporate synthesized chemosensors *via* chemical grafting, and the final functionalized cellulose was responsive to cyanide anions in the aqueous solutions [79]. Ozone was used to create hydroperoxides on the rayon fibers, and the ozone-treated fibers were immediately placed in AA aqueous solution containing Fe(II) ammonium sulfate hyxahydrate salt, which formed a redox initiator. The grafting process was performed in a nitrogen atmosphere, in sealed glass ampoules for 60 min, with pH adjusted to 1.5 [80].

Despite being modified by organic molecules, cellulose derivatives can also be chemically incorporated with inorganic particles. Peng *et al.* synthesized interfacially active and magnetically responsive nanoparticles by grafting BriB functionalized EC onto amine functionalized Fe_3_O_4_ nanoparticles for multiphase separation applications [81]. Gaharwar *et al.* developed magnetic nanoparticles encapsulated within HPC by a facile two-step approach [82]. Magnetic nanoparticles (MNP) were prepared via a controlled chemical co-precipitation approach, and were modified by (3-aminopropyl) trimethoxysilane (APTMS). Partially oxidized HPC aqueous solution was mixed with saturated NaIO_4_ aqueous solution in the dark at room temperature for 18 h, and then MNP/APTMS solution was added and the mixture was stirred for 12 h to get the final magnetic nanoparticles with thermal responsive shells.

### 2.2. Physical Incorporating/Blending

Cellulose is widely utilized to fabricate polymer blends and composites due to its wide abundance, renewable, environmentally benign nature, and its outstanding mechanical properties [17,18,19,83]. In the fabrication process of composite materials containing cellulose, cellulose plays an important role as matrices, fillers, or coatings/shells.

#### 2.2.1. Matrices

**Figure 14:**
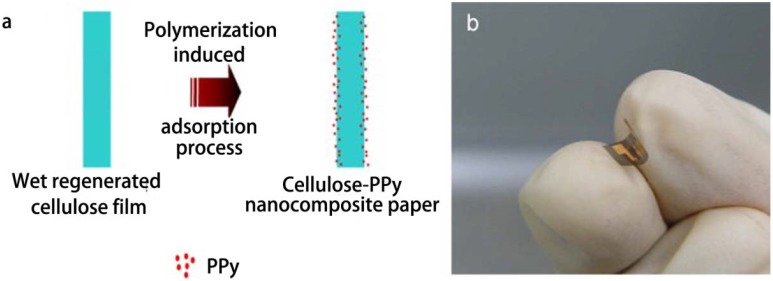
(**a**) Schematic of cellulose-PPy nanocomposite fabrication process; (**b**) Fabricated flexible humidity and temperature sensor. Reprinted with permission from [92]. Copyright 2010 Elsevier.

Cellulose membranes/films are used to prepare “smart” materials as supporters. Zhang and Wu reported a method of dispersing responsive nanoparticles in EC membrane by casting formation [84,85]. Glucose-responsive membranes were produced by dispersing poly(NIPAm-*co*-MAA) nanoparticles, glucose oxidase, and/or catalase in EC membrane using the same casting method. An organic vapors-sensitive composite film comprising CA and a representative compound (1-n-butyl-2,3-dimethylimidazolium hexafluorophosphate, [BM_2_Im][PF_6_]) was developed by Regmi *et al.* [86]. Films were prepared using a solvent precipitation method. To the CA and [BM_2_Im][PF_6_] solution, anhydrous heptane was added dropwise under stirring, and the mixture was then transferred to PTFE beaker and cleaned quartz crystal for *in situ* monitoring organic vapors. Cholesteryl oleyl carbonate (COC), having a high temperature coefficient of the selective reflectance near room temperature, was embedded in cellulose nitrate (CN) membranes using vacuum filtration method or absorption method to produce temperature-responsive membranes [87,88]. In the vacuum filtration method, a CN membrane was mounted on a stainless steel filter holder and COC dissolved in chloroform was filtrated using reduced pressure, and was dried to obtain the COC-embedded membrane. In the absorption method, COC was absorbed onto CN membranes by immersing in chloroform solution. Liquid crystal (LC)-embedded CA and CN membranes with temperature-responsive properties were also produced by absorption of *n*-heptyl cyanobiphenyl (K21), a thermotropic LC [89]. To produce LC-embedded CA membranes, CA sheets were soaked in K21 previously warmed to above the nematic-isotropic phase transition temperature, above which LC molecules are at an isotropic phase and can therefore move around freely and be distributed more easily within the membrane pores. To fabricate CN membranes embedded with LC, CN membranes were set on a sintered glass funnel, and extra K21 (more than the porosity of the membrane), warmed up to 46 °C, was passed through the CN membrane by means of vacuum. An enantioselective-controlled drug delivery membrane system for selective release of the required (*S*)-enantiomer in response to pH stimuli was developed by the phase inversion method [90]. The recognition system was obtained from a nanoparticle-on-microsphere molecularly imprinted polymer (NOM-MIP) with a multifunctional chiral cinchona anchor. Prepared NOM-MIP beads with or without being drug loaded were thoroughly mixed with cellulose NMMO solution, and the mixture was poured into a flat-bottomed glass Petri dish, then transferred into a beaker containing distilled water and left for 12 h. Finally, the membrane was recovered and dried overnight at room temperature. Series fluorescence sensors for trace monitoring of dissolved ammonia were developed by Waich *et al.* based on cellulose esters membranes [91]. Eosin and synthesized 2′,7′-dichlorofluorescein methylester (DCF) were used as sensing dyes. Bulk sensing membranes were prepared by spreading cocktails containing cellulose esters and dyes dissolved in acetone onto polyester films and then covering with a thin silicone layer. Particle membranes were prepared by resuspending nanospheres of cellulose esters and dyes in silicon solution in an ultrasonic bath, and spreading the suspension onto polyester films, then covering another silicone layer. Polymerization-induced adsorption (PIA) method was adopted by Mahadeva *et al.*, to produce cellulose-polypyrrole nanocomposite paper as a flexible humidity and temperature sensor (Figure 14) [92]. PIA is a technique of processing ultra-thin polymer films, involving immersion of substrate into polymerization solution, leading to the growth of polymer chains on the substrate surface [93]. CuCl_2_·H_2_O was added dropwise into a pyrrole solution for polymerization, and a very dilute polymerization solution of polypyrrole (PPy) was obtained by filtering reaction mixture to remove the bulk PPy. The filtered solution was added into a glass Petri dish and then the wet RC films were immersed and left for polymerization. *In situ* polymerized PPy was slowly formed and continuously deposited on the cellulose surface and monomer pyrrole was polymerized after being adsorbed on the cellulose.

Cellulose, with asymmetric crystalline structures displaying inhomogeneous deformation of train gradients associated with the piezoelectric response due to an applied electric field [94], has been discovered to be an electro-active polymer. Cellulose paper has many advantages in terms of large strains in response to an electric stimulus, ease of processing, good mechanical properties, low density, low cost, biodegradability, low actuation voltage, and low power consumption as sensors and actuators [94,95], termed electro-active paper (EAPap). Kim *et al.* [95] and Pandey *et al.* [96] have reviewed some methods to fabricate EAPap using regenerated cellulose films, PIA modified cellulose films, multi-walled carbon nanotubes functionalized cellulose films, cellulose–chitosan blend films, *etc.* Cellulose and sodium alginate mixed films were regenerated from NaOH/urea solutions and deposited with thin gold electrodes to fabricate EAPap [97]. Cellulose derivatives were also applied. Commercial cellophane, a well-known cellulose film made with cellulose xanthate, was used as a cellulose film for EAPap actuator [98]. Li *et al.* developed an electrospun fullernol–cellulose biocompatible actuator with much lower power consumption and larger electromechanical displacement in comparison with a pure cellulose acetate actuator [99]. Kunchornsup and Sirivat developed a physically cross-linked cellulosic gel using 1-butyl-3-methylimidazolium chloride (BMIMCI) ionic liquid [100]. Cellulose was dissolved in BMIMCI and DMAc was added as a plasticizer and a co-solvent. The mixture was degassed and casted into a mold and kept under a vacuum for 12 h, then curing at ambient conditions for 24 h to get the physically cross-linked cellulosic gel, which was promising for actuator applications over other existing dielectric elastomers.

Using EC as polymeric support materials for electrospun nanofibrous materials as highly responsive fluorescence quenching-based copper and mercury sensitive chemosensor was reported [101,102]. EC, plasticizer, and sensitive dyes were dissolved in THF to produce polymeric precursor solution, which was electrospun into fibers or spread onto a polyester support to produce thin films. The pH-indicator Methyl Red was applied onto cellulosic textile fabrics using both a conventional dyeing and a sol-gel technique [103]. All conventional dyeing methods were performed in a Mathis Labomat BFA-8 lab dyeing machine using a direct dyeing process for cotton. In the sol-gel technique, the organosilicon precursor glycidoxypropyltrimethoxysilane (GPTMS) was applied because the size of standard pH-indicators was too small for a permanent entrapment in a sol-gel matrix. GPTMS was firstly reacted with Methyl Red in ethanol at 70 °C for 6 h in the presence of a catalytic amount of HCl. Hydrolysis and condensation reactions took place with water added in the mixture leading to the hybrid sol-gel materials. The cotton samples were impregnated with these sols by passing them through a two-roll laboratory padder four times in a nip pressure of 4 bar and twice at 2 bar. After dyeing, the fabrics were cured in an electric laboratory oven.

Carboxymethylcellulose esters (acetate ester, acetate butyrate ester, acetate propionate acetate) mixed with drugs were compressed into tablets to get the pH-responsive drug delivery systems [104], and the HPC mixed with sodium alginate microbeads loaded with heparin that were prepared by suspending emulsified heparin in aqueous mixture of HPC and sodium alginate and that were physically crosslinked by CaCl_2_ showed temperature-responsiveness [105]. Hydroxypropyl methyl cellulose (HPMC) aqueous solution suspended with pectin, drugs, and calcium carbonate was emulsified with light mineral oil, and the obtained microbeads were coated with EC in a fluidized bed by spraying the EC solution in acetone on the gel beads using a spray gun [106,107]. These drug delivery systems were pH sensitive, where HPMC played an important role as a matrix in loading the drugs, and EC as protecting coatings in prolonging the drug-release periods. Drugs dispersed in EC matrix were coated with nano-sized PNIPAm gels, and the obtained formulation was thermosensitive controlled-release [108].

Cellulose has also been widely used as matrix for inorganic materials. Chemical vapor deposition (CVD) of a thin titanium dioxide (TiO_2_) film on lightweight native nanocellulose aerogels offers a novel type of functional material that shows photoswitching between water-superabsorbent and water-repellent states [109]. Aqueous gels of long and entangled native cellulose I nanofibrils were prepared using a method by combining refining, enzymatic treatment, and high-pressure homogenization [110], and the prepared aqueous gel was used to prepare nanocellulose aerogels by vacuum freeze-drying method [111]. The nanocellulose aerogels were coated with TiO_2_ in an atomic layer deposition reactor by CVD, followed by reacting with the precursor titanium isopropoxide, Ti(C_3_H_7_O)_4_, at 190 °C and 1–5 kPa for 2 h. In the end, the sample was purged with nitrogen at the reaction temperature for 2 h to remove any unreacted species, and then cooled in nitrogen flow to room temperature for unloading. The same CVD procedure was performed for filter papers and nanocellulose films by film-casting of aqueous nanocellulose aerogels. Magnetic nanoparticles (Fe_3_O_4_) were incorporated into bacterial cellulose matrix by ammonia gas-enhancing *in situ* co-precipitation method to produce magnetically responsive bacterial cellulose sheets [112]. Tan *et al.* developed a controllable aggregation and reversible pH sensitivity of gold nanoparticles regulated by CMC [29]. Chlorauric acid aqueous solution was added to CMC aqueous solution and the mixture was kept for 12 h at 110 °C under constant stirring to obtain colloidal solution, in which cysteamine hydrochloride aqueous solution was added. The mixture solution was further stirred for 2 days at room temperature to obtain the dispersion of Au/CMC assembly.

#### 2.2.2. Fillers

**Figure 15:**
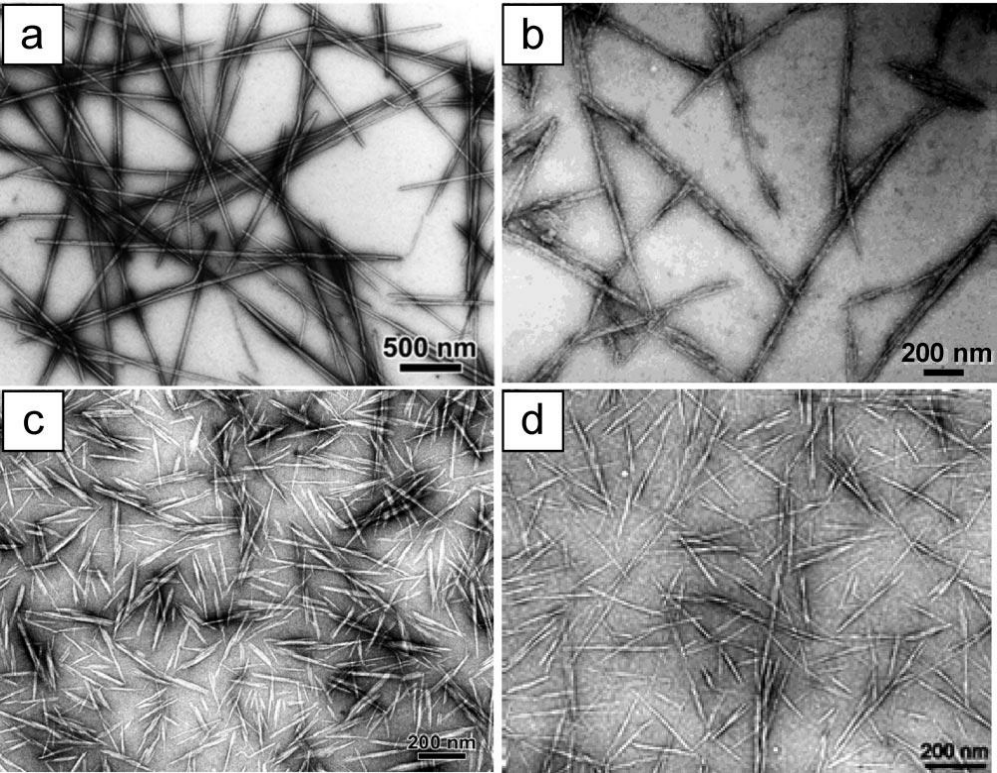
TEM images of dried dispersion of cellulose nanocrystals derived from (**a**) tunicate; (**b**) bacterial; (**c**) ramie; (**d**) sisal. Reprinted with permission from [69]. Copyright 2010 American Chemical Society.

CNC has high mechanical strength and elastic modulus values, which make it an ideal component in many nanocomposites [7,17], arising from interactions between the crystalline and amorphous regions, as well as from the properties of these regions themselves. CNCs can be isolated from a variety of renewable sources, including plants (such as wood, cotton, or wheat/rice straw), as well as from bacterial sources or animal (e.g., tunicates) tissue through acid hydrolysis, and the obtained rod-like CNCs have a morphology and crystallinity similar to the original cellulose fibers [69,113]. Figure 15 shows some examples of such elements.

CNCs have been widely used as reinforcement in shape-memory materials, which have the capability of changing their shape upon an external stimulus. Polyurethane (PU) is an important shape-memory material, containing segments of high polarity with a high concentration of urethane and urea bonds on each polymer chain, therefore exhibiting a high thermal transition temperature duo to their high intermolecular interaction [114,115,116]. Shape-memory CNCs/PU composites were fabricated with CNCs prepared from crystalline cellulose originated from different sources by hydrolysis treatments [117,118,119,120,121]. CNCs were firstly dispersed in DMF at a specific concentration to form homogeneous suspensions, and the suspensions were mixed with PU solutions in DMF, and the nanocomposites materials were solidified in Teflon mold for 24 h at 60 °C. Auad *et al.* reported using polyaniline-modified cellulose nanofibrils as reinforcement to prepare thermal-responsive shape-memory composites with reducing electrical resistivity [122]. Polyaniline-modified CNCs were prepared by *in situ* polymerization of polyaniline onto the CNCs surfaces. And the polyaniline-modified CNCs were redispersed in DMF and mixed with PU. Composite films were prepared by casting and further solvent evaporation. New biomimetic, stimuli-responsive mechanically adaptive nanocomposites, which changed their mechanical properties upon exposure to water and displayed a water-activated shape-memory effect, were prepared by dispersing cellulose nanowhisker organogels into a rubbery PU matrix [123]. CNCs organogels were fabricated from aqueous dispersions using a solvent-exchange sol-gel process where gelation was induced through addition of a water-miscible nonsolvent (acetone) to the CNCs dispersion. The CNC organogels were then added in DMF solution of PU and dissolved, and the mixture was cast into Teflon dishes to obtain the CNCs/PU nanocomposites.

The development of a new class of mechanically adaptive nanocomposites with dispersed CNCs has been inspired by biological creatures such as sea cucumbers, which have the ability to reversibly change the stiffness of their dermits. Owing to the abundance of surface hydroxyl groups, CNCs display strong interactions between themselves, causing the evenly dispersed percolating nanocomposites to display a high stiffness. The nanocrystal–nanocrystal interactions can be largely switched off by the introduction of a chemical regulator that allows for competitive hydrogen bonding, resulting in a significant decrease in the stiffness of the material [113]. Nanocomposites based on ethyleneoxide/epichlorohydrin copolymer (EO-EPI), poly(butyl methacrylate) (PBMA), and PVAc were developed [124,125,126]. CNCs were dispersed in the polymer solutions and mixed in DMF, and the nanocomposite films were obtained by solution casting methods. These materials showed significant decreases of tensile storage moduli upon exposure to physiological conditions, water, or high temperatures. Nanocomposites based on poly(styrene-*co*-butadiene) (SBR) and polybutadiene (PBD) were prepared by dispersing sulfonated tunicate whisker organogels in the polymer matrices, which showed water-triggered modulus changes [127]. Carboxylated CNCs and amine-functionalized CNCs dispersed in PVAc polymer matrices showed pH-responsive modulus changes due to the hydrogen-bonding changes of carboxylates and amines *via* protonation and deprotonation with pH changes [72].

Cellulose nanofibrils [128] and HPC [51] were used as pore-forming agents in “smart” hydrogels to improve the swelling capacities of the hydrogels. Cellulose nanofibrils were obtained through acid hydrolysis reaction using HCl from cotton fibers, and were added to the reaction mixture of chitosan and AA to form the chitosan-graft-poly(acrylic acid)/cellulose nanofibrils hydrogel composites via free radical copolymerization. HPC was used to modified poly[(*N*-[3-(dimethylaminopropyl)] methacrylamide)-*co*-acrylamide] hydrogels.

#### 2.2.3. Coatings/Shells

Cellulose derivatives, such as EC and HPC, have long been utilized in the pharmaceutical industry as coatings and shells of microspheres [9,129,130,131,132,133,134]. These derivatives were also used to fabricate stimuli-responsive materials as coatings and shells.

EC and cellulose acetate phthalate were used to coat drugs, which were dispersed in stimuli-responsive matrices, as protecting shells [106,135,136]. EC was also applied as a protecting film to fabricate humidity sensors made of lanthanum ferrite/polymer quaternary acrylic resin [137]. A fluorescent amphiphilic cellulose nanoaggregate-sensing system was designed by Wang *et al.* and applied in detecting explosives in aqueous solution [138]. The amphiphilic self-associated cellulose derivatives (HMHEC) were synthesized by introducing hydrophobic moieties into the backbone of HEC. The HMHEC could self-assemble to nano-micelles with a hydrophobic inner core and a hydrophilic outer shell in aqueous solution, and the hydrophobic poly(9,9-dioctylfluorene) (PFO) was loaded in the cores of the nano-micelles. The hydrophilic outer shells formed by the polysaccharide main-chain ensured the dispersion of the aggregates in water and also acted as a protector. 

Cellulose derivatives were applied as coatings to produce magnetic-responsive materials with magnetic nanoparticles cores [82,139]. Iron suspension in HNO_3_ aqueous solution was prepared by emulsifying with sodium dodecyl sulfate and stabilizing with polyethylene glycol. EC dissolved in benzene/ethanol was added to the iron solution, and the two phases were heated and stirred vigorously. The organic solvent was completely evaporated using rotary evaporation to obtain an aqueous suspension of pure EC nanoparticles. The composite core/shell nanoparticles were cleaned by repeated magnetic separation and re-dispersion in an aqueous medium. The drug, 5-fluorouracil, was loaded by single surface adsorption and entrapment procedure [139].

## 3. Properties and Applications

### 3.1. Drug Delivery Systems and Biomaterials

Drug delivery systems with response to pH, temperature, redox potential, light, and magnetic fields, *etc.* are able to promote drug release, to reach specific intracellular locations, or to target tissues. “Smart” drug delivery systems have been intensively studied and reviewed in recent years [140,141,142,143,144,145,146,147,148]. Polymeric systems based on cellulose with responsive behaviors have showed unique properties such as biocompatibility, biodegradability, and biological functions and have been exploited by many researchers. Different forms of polymeric systems based on cellulose for drug delivery and biomaterials are discussed as follows.

#### 3.1.1. Aggregates and Hydrogels

Stimuli-induced self-assembly and post-assembly triggering strategies provide an alternative approach for the manipulation of self-assembled architectures of synthetic polymeric aggregates in drug delivery systems. The assembly of polymeric aggregates results in transmittance and hydrodynamic radius (*R*_h_) changes, and drugs loaded in the assembled polymeric aggregates can be released while they disassemble in the stimuli of surrounding environment, such as temperature and pH.

**Figure 16:**
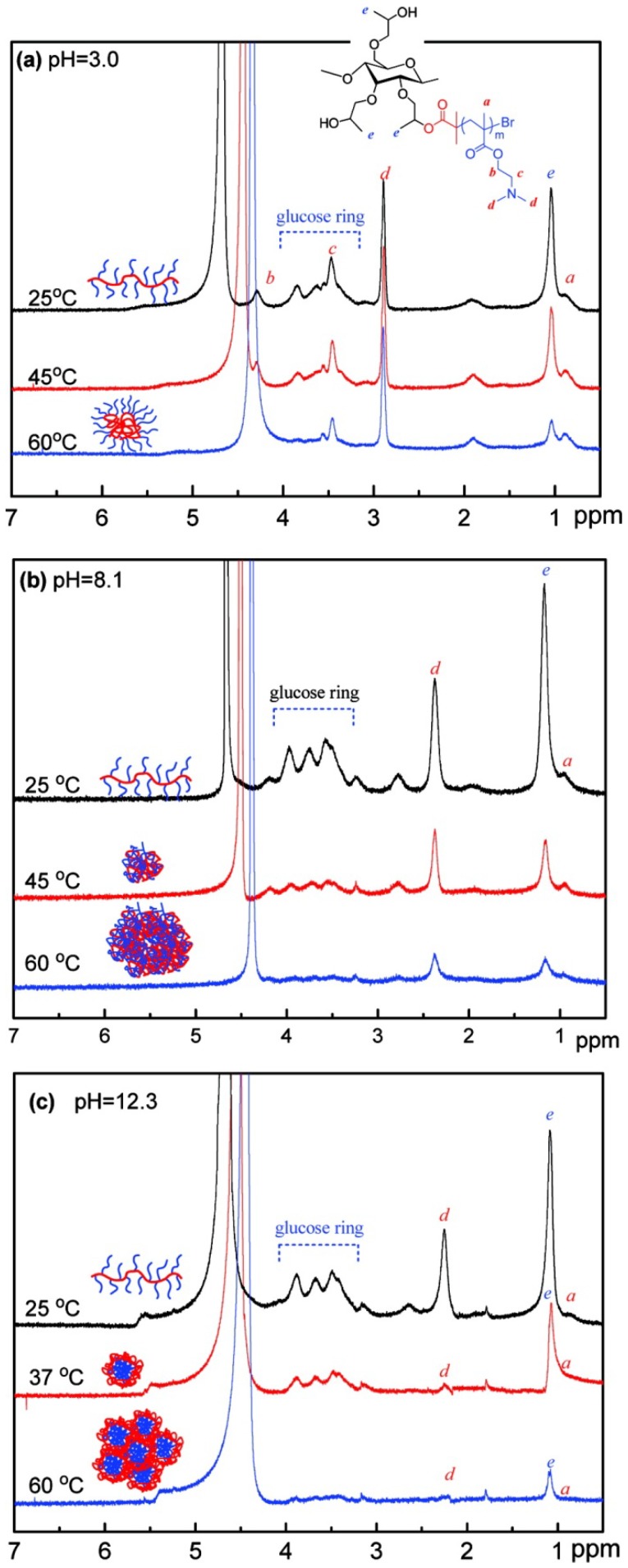
Temperature dependence of ^1^H NMR spectra of HPC-*g*-PDMAEMA solutions in D_2_O at pH (**a**) 3.0; (**b**) 8.1; and (**c**) 12.3. Reprinted with permission from [53]. Copyright 2010 American Chemical Society.

**Figure 17:**
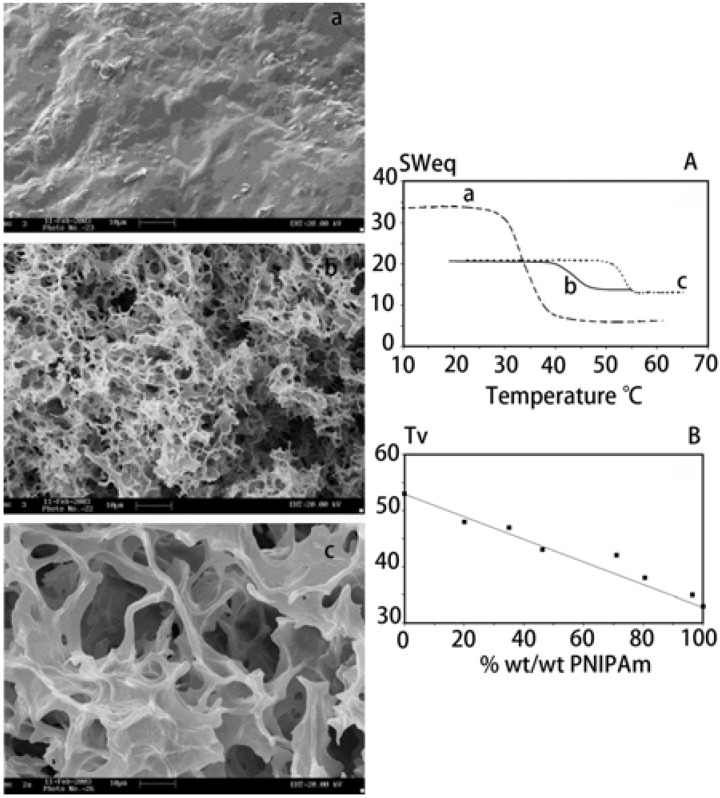
Left: SEM images of freeze-dried gels; Right: (**A**) Equilibrium swelling degree (SWeq) *vs.* temperature; (**B**) Temperature of the volume phase transition *T*_v_ for HPC/PNIPAm-IPN having different composition. (**a**) PNIPAm; (**b**) HPC/PNIPAm interpenetrated network (composition of 53.7/46.3 wt/wt); (**c**) HPC. Reprinted with permission from [56]. Copyright 2004 Elsevier.

The temperature- and pH-sensitive characteristic of cellulose-*g*-PDMAEMA was examined by UV detection and dynamic light scattering (DLS) by Sui *et al.* [66]. The LCST of aqueous cellulose-*g*-PDMAEMA solution was measured to be 42 °C. If the temperature remained below this number, the solution was transparent and the *R*_h_ value increased slightly with temperature increase (25–40 °C). If, however, the temperature was raised to the range of 42–55 °C, the solution became opaque and the *R*_h_ value increased drastically. At a low temperature, the cellulose-*g*-PDMAEMA copolymer chains existed in random coil conformation owing to the hydrogen-bonding interactions between the copolymer and water molecules. As the temperature increased to LCST, polymer chains shrank into a globular structure because of the hydrophobic interactions between *N*,*N*-dimethylaminoethyl groups. The cellulose-*g*-PDMAEMA was dissolved in HCl aqueous media with pH 2.0 at room temperature and was precipitated in aqueous media with pH increase to 12.0. At low pH, the PDMAEMA chains were entirely protonated and highly stretched along the radial direction because of the geometrical constraint and the electrostatic repulsion between polymer chains. While at high pH, PDEAEMA chains gradually shrank and precipitated from the solution due to the deprotonation of amine groups. HEC-g-(PNIPAm and PAA) exhibited similar thermo- and pH-responsive characteristic with LCST of 34 °C and p*K*_a_ of 4.6 [59]. Ma *et al.* illustrated the intermolecular/intramolecular interactions changes with temperature and/or pH changes of HPC-*g*-PDEAEMA [53] and HPC-*g*-P4VP [54] by liquid ^1^H NMR (Figure 16). For HPC-*g*-PDEAEMA at low pH value, e.g., pH 3.0, the intensity of the peaks for HPC backbone (*e* and the glucose ring in Figure 17a) decreased at 45 °C (near the LCST of HPC), compared to those peaks at 25 °C and disappeared with the further rising of temperature up to 60 °C, whereas the peaks of PDMAEMA side chains remained unchanged in all the experimental temperatures (*a*, *b*, *c*, *d* in Figure 16a). It is known that PDMAEMA (p*K*_a_ ≈ 8.0) is a weak polyelectrolyte and can be protonated in an acidic aqueous solution. The LCST of PDMAEMA shifted to a higher temperature with the decrease in pH due to the protonation of PDMAEMA chain, leading to the increase in the electrostatic repulsive force and prevention of the phase separation. Therefore, when the HPC-*g*-PDMAEMA acid aqueous solutions were heated, the HPC backbone collapsed to form the core of the micelles and stabilized by the hydrophilic PDMAEMA side chains as the shell. When the solution pH of HPC-*g*-PDMAEMA copolymer were changed to 8.1, the intensity of the peaks for both HPC backbone and PDMAEMA side chain decreased simultaneously upon heating (Figure 16b), which indicated that both the HPC backbone and the PDMAEMA side chain collapsed with increasing temperature. At even higher pH value, e.g., pH 12.3, the intensity of the peaks for PDMAEMA side chain disappeared at 37 °C, whereas the intensity of the peaks of HPC backbone decreased slightly (Figure 16c), indicating that PDMAEMA side chain aggregated to form the core of micelles and the HPC backbone mainly as the shell to stabilize the micelles. At an even higher temperature above the LCST of HPC, e.g., 60 °C, the peaks for both HPC and PDMAEMA disappeared, which indicated the shell HPC aggregated and lost the ability to stabilize the micelles. Moreover, the results of transmittance and *R*_h_ conformed to the ^1^H NMR results. The ^1^H NMR results for HPC-*g*-P4VP showed similar changes according to the pH or temperature. Yuan *et al.* prepared amphiphilic EC brush polymers with mono and dual side chains, which showed promising properties with dual temperature and pH response according to the results of UV, DLS, and transmission electron microscopy (TEM) [62].

Stimuli-responsive hydrogels, which are able to swell or shrink as a function of external stimuli, have recently gained a great deal of attention, especially for their use in biomedical applications due to their unique properties such as biocompatibility, biodegradability, and biological functionality [26,27]. The mechanism of hydrogels’ swelling and shrinking is similar to that of aggregate assembly: stimulus-induced intermolecular and intramolecular hydrogen-bonding changes. Drugs loaded in the hydrogels can be released while they swell to looser structures due to environmental changes in the vicinity.

Changes of *R*_h_ and transmittance can also be detected for cellulose-based “smart” microgels in response to temperature, pH, or MAA [32,33,49,50]. Swell properties of “smart” hydrogels and the loaded drug-release properties are frequently characterized. Marsano *et al.* reported a well-defined porous structure of HPC and PNIPAm interpenetrated network (Figure 17 (left panel)) [56]. The presence of HPC conferred a much higher porosity to the IPN than that of neat PNIPAm. The trend equilibrium swelling degree (SW_eq_) *vs.* temperature was reproducible even in cooling course in the examined range of temperature. Volume transition temperature (*T*_v_) was taken as the temperature corresponding to the inflection point of the curve SW_eq_ *vs.* temperature. The differences of swelling behaviors of HPC, IPN, and PNIPAm (Figure 17 (right panel)) were in accordance with the differences of structures and could be explained on this basis. The swelling properties could be influenced by the compositions of the hydrogels, which also had effects on the response rates to stimulus [57]. The biocompatibility of “smart” hydrogels based on cellulose was confirmed by cell viability tests by Xu *et al.* [55] and Tan *et al.* [58]. Hydrogels responsive to temperature or pH are usually studied as drug delivery systems. BSA, dextran, insulin, oxaliplatin, and ketoprofen were applied as drug models to evaluate the release properties of these “smart” hydrogels in response to temperature and pH [32,33,45,50,55,149]. Redox-responsive hydrogels also have potential applications in controlled drug release, because redox is important in biology and can be used in redox switches and signaling [58]. A pH-responsive CMC-based hydrogel membranes incorporating acrylate was prepared for drug delivery [35]. The diffusion of salicylic acid demonstrated that the polymer could be used as an enteric coating polymer to protect the stomach wall from the harmful effect of drugs or to protect drugs in a dosage from degradation in the gastric fluid.

#### 3.1.2. Microcapsules and Nanoparticles 

**Figure 18:**
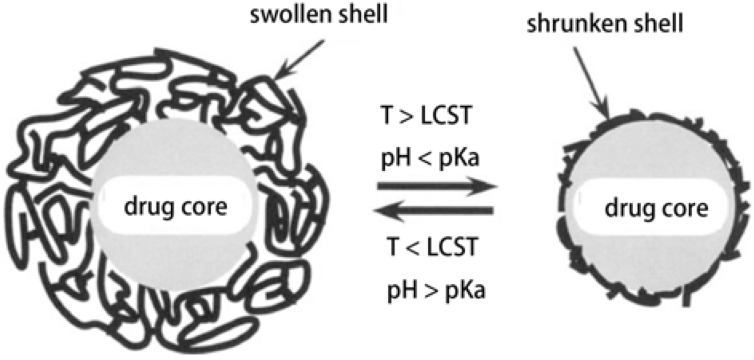
Schematic diagram showing ideal particle structure of drug carriers with temperature- and pH-responsive shells [108].

Polymeric materials are often used to control the release rate of drugs out of the pharmaceutical dosage form, with the drugs either directly embedded within a macromolecular network (matrix system) or surrounded by a polymeric membrane (film coating, reservoir system). In the latter case, the diffusion of drugs through the macromolecular shell can control the resulting release kinetics. Heparin was encapsulated in alginate-HPC microbeads, with average diameters of *ca*. 3.0 ± 1.6 μm and the heparin release efficiency was lower at elevated temperatures than at room temperature [105]. The observed effect reflected the morphological changes of the thermosensitive beads on heating. Above LCST, the more compact conformation of HPC chains lead to less rotational freedom of heparin macromolecules, thus the heparin release was slower. Slow heparin release from the gel at the physiological temperature constituted the real advantage of the system. Fast initial stage followed by the long-term slow steady release allowed for attaining quickly the necessary concentration of the delivered drug and maintaining this level for the time sufficient to assure its therapeutic effect. Fang and Cathala prepared microparticles containing CMC by a microfluidic approach and the microparticles loaded with BSA showed pH-responsive swelling and release properties [47]. EC blended with or grafted with responsive polymers were used as coating materials to prepare coated particles with drug cores [61,108,135]. Drugs release behaviors are dominated by the shell swelling properties in response to stimulus (Figure 18).

Gold nanoparticles have attracted increasing interest in recent years because of their unusual properties and potential applications in biomaterials and medicine. Modification of gold nanoparticles with smart polymers can tailor the dispersibility with stimuli-responsive properties, which are of great interest in intelligent drug delivery. CMC modified gold nanoparticles showed pH-responsive aggregation properties and could offer versatile technological and medicinal applications [29]. Magnetic-responsive drug delivery systems are widely used to trigger drug release at target sites, and can also be utilized to concentrate the drug-specific responsive carriers. Magnetic cores decorated by EC can improve the biocompatibility and 5-fluorouracil loaded in the nanoparticles could be controllably released for cancer targeting [139]. Gaharwar *et al.* used HPC as coating materials to prepare magnetic particles [82]. Such a unique combination of thermo-responsivity and magnetism could open up novel prospects in the field of nanomedical applications such as remote-controlled drug carriers.

#### 3.1.3. Membranes

**Figure 19:**
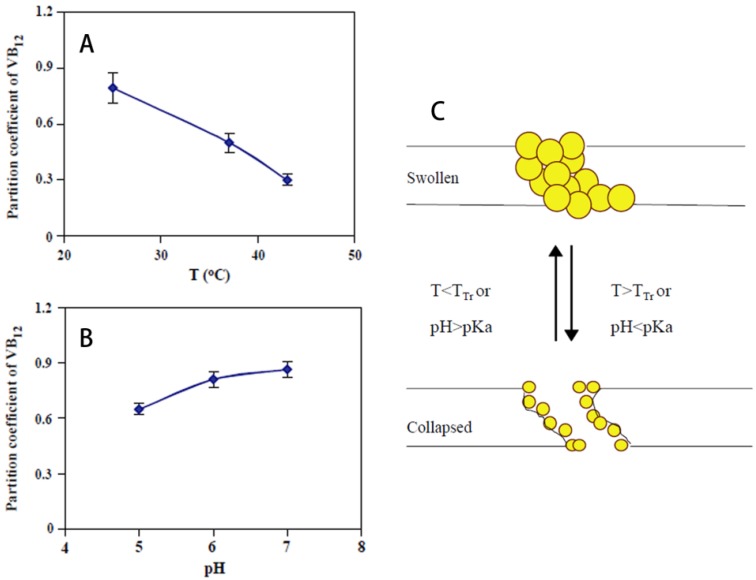
Partition coefficient of vitamin B12 into a composite membrane with 30 wt % of 1:0.4 particles (**A**) In 0.1 mM KCl at varied temperature; (**B**) In 0.15 M PBS with varied pH values at 28 °C; (**C**) Schematic illustration of the permeation model for a composite membrane containing temperature- and pH-responsive nanoparticles. Reprinted with permission from [84]. Copyright 2003 Elsevier.

**Figure 20:**
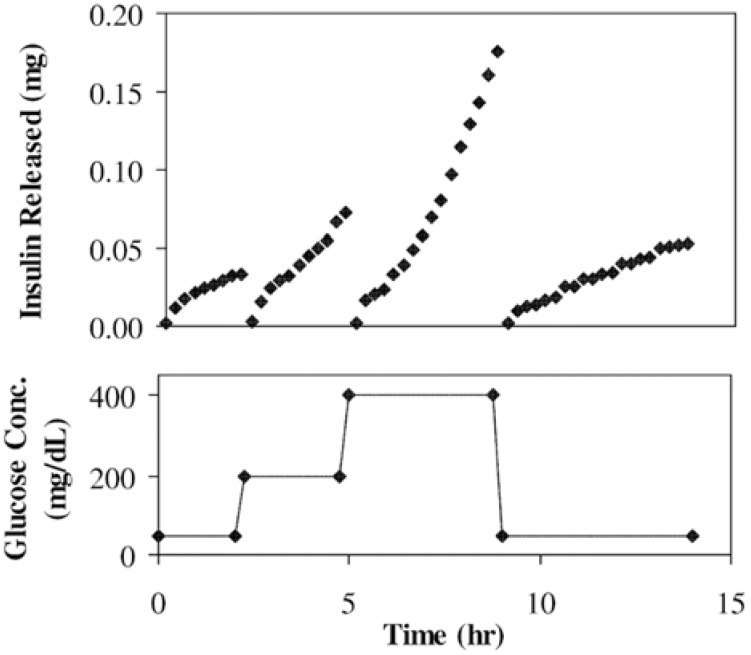
Profiles of insulin delivery across a membrane in response to glucose steps (50 to 200 to 400 to 50 mg/dL) in pH 7.4 PBS (10 mM/0.15 M NaCl) at 37 °C. The membrane consisted of 1.5 mg of GOD/0.43 mg of catalase and 35 wt % of the nanoparticles with a NIPAm:MAA molar ratio of 1:1. Reprinted with permission from [85]. Copyright 2002 Elsevier.

Membranes with stimuli-responsive materials can change their pore size according to the environmental conditions [63,150], thus drug delivery systems made with smart membranes can release drugs in response to stimulus by diffusion through the membranes. CMC esters mixed with aspirin were pressed to the tablet membrane, which showed zero-order release of the drug with pH-responsive properties [104]. Poly(NIPAm-*g*-AA) nanoparticles dispersed in EC membranes showed pH- and temperature-responsive permeation of vitamin B_12_, and the partition coefficient decreased with temperature increase and with pH decrease, the mechanism of which was probably the change of pore size of the membrane due to the deformation of poly(NIPAm-*g*-AA) (Figure 19) [84]. The same research group reported that poly(NIPAm-*g*-MAA) nanoparticles dispersed in EC membranes showed glucose-sensitive properties (Figure 20) [85]. The results indicated that modulated insulin permeation by glucose concentration could be achieved in a discontinuous buffered condition. Such reversible glucose-responsiveness was ascribed to the reversibility of swelling and shrinking of the nanoparticles in response to changes of pH. CN and CA membranes embedded with liquid crystal molecules also showed temperature-responsive drug permeation characteristics [88,89]. Suedee *et al.*, prepared an MIP incorporated cellulose membrane for enantioselective-controlled delivery of racemic drugs with pH-responsiveness [90]. (*S*)-omeprazole was used as an imprinting molecule conferring stereoselectivity upon the polymers. The ability of the prepared recognition polymers to selectively rebind (*S*)-omeprazole was evident at different pH levels (the highest being at pH 7.4). The partial selective-release phenomenon of the (*S*)-enantiomer in MIP-containing composite cellulose membranes with increased vehicular racemic omeprazole concentrations was highly pH-dependent. Cinchona-bonded polymers imprinted with (*S*)-omeprazole could recognize the moldable contact site of (*S*)-omeprazole independently of its chirality, and this was responsible for the delivery of (*S*)-enantiomer from racemic omeprazole.

### 3.2. Hydrogels

Hydrogels, due to the abundant hydrophilic groups on the chains and slightly crosslinked structure, can absorb large amounts of water and release the absorbed water in dry conditions. Besides their applications in drug delivery, hydrogels have wide potential applications in the fields of food, biomaterials, agriculture, *etc.* The preparations and applications of hydrogels based on cellulose have been reviewed by other researchers [27,151], thus herein we focus on the stimuli-responsive hydrogels based on cellulose.

**Figure 21:**
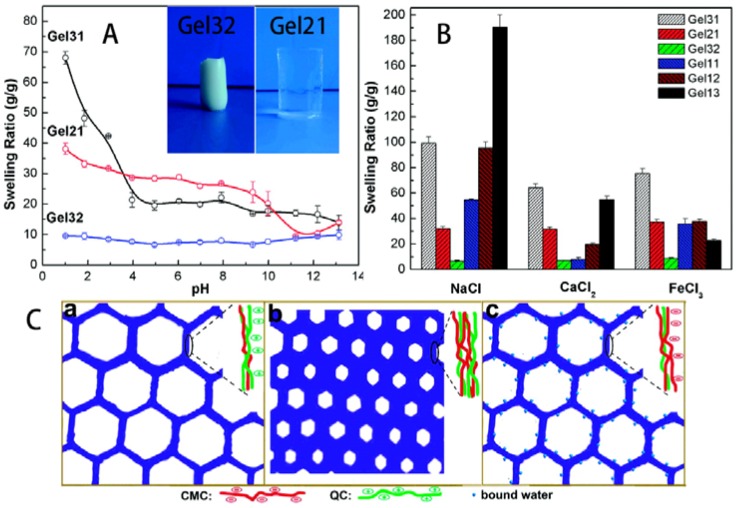
(**A**) Effects of pH on the swelling behaviors of QC/CMC hydrogels in buffer solutions; (**B**) Swelling ratio of hydrogels in different salt solutions (0.01 M): NaCl, CaCl_2_, and FeCl_3_; and (**C**) Schematic illustrations of the structures of QC/CMC hydrogels: (**a**) Gel31; (**b**) Gel32; and (**c**) Gel13. Reprinted with permission from [44]. Copyright 2011 American Chemical Society.

“Smart” hydrogels based on cellulose are usually made of HPC and CMC, not only because of the water-solubility of these derivatives, but also because of the temperature-responsive properties of HPC and pH-responsive properties of CMC. These hydrogels possessed temperature- or pH-responsive characteristics as their cellulose ingredients [38,46,57]. Apart from that, these hydrogels were salt-responsive in aqueous media. The higher the concentration, and the higher the chelating ability of the salts, the less water the hydrogels could uptake. The increase in the ionic strength reduced the difference in the concentration of movable ions between the polymer matrix and the external solution and led to an immediate contraction of gel. The decreasing was more significant to ions with higher valence, like Ca^2+^, Mg^2+^, Al^3+^, arising from the complex formation ability of the hydrophilic groups, including intramolecular and intermolecular complex formations, or because one multivalent ion was able to neutralize several charges inside the hydrogels [38,46,128]. Salmawi and Ibrahim reported that clay mixed with CMC reduced the water uptake capacity of the prepared hydrogels [48], while another research group reported that the water uptake capacity could be improved by adding a suitable amount of rectorite, yet excessive rectorite induced the reduction in water absorption [36]. The superabsorbent nanocomposites based on CMC and rectorite also showed saline, pH, and organic solvent responsive. Using CNC to prepare “smart” hydrogels could not only improve the swell capacity but also improve the mechanical strength of the hydrogels [41,128]. Chang *et al.* prepared ampholytic hydrogels with pH- and salt-responsive properties by crosslinking QC and CMC with epichlorohydrin (Figure 21) [44]. QC in the hydrogels played an important role in the domination of the amount of charges, leading to high pH sensitivity, whereas CMC mainly contributed to the increasing of equilibrium swelling ratio of the hydrogels. The electrostatic repulsion between the hydrogel backbone (–(CH_3_)_3_N^+^/–COO–) and the pH solution (H^+^/OH^−^), the electrostatic repulsion among cellulose chains, electrostatic screening between the hydrogel backbone and the pH solution, and electrostatic screening between the –(CH_3_)_3_N^+^ and –COO– groups all led to pH responses of the hydrogels (Figure 21A). In NaCl, CaCl_2_, and FeCl_3_ solutions, the swelling ratio of all hydrogel samples mostly decreased with the increase of salt concentration. However, the swelling ratios of the hydrogel samples were different in varying salt solutions (Figure 21B). Generally, expansion of an ionic hydrogel is related to a balance between the osmotic pressure (driven by ions inside and outside the hydrogel), polymer-solvent interactions, and elastic retractile force of polymer. The osmotic pressure in gels of polyelectrolyte and the repulsive force between fixed charges play an important role in the expansion of the hydrogel. Thus, the chemical compositions of the hydrogels could influence water absorption capacity (Figure 21C). Gel31 had relatively higher swelling ratio as a result of the excess positive charges fixed in the hydrogel networks (Figure 21C(a)). Gel32 had minimum swelling ratio due to the absence of free charges in the hydrogel network (Figure 21C(b)). However, excess negative charges were fixed in the network of Gel13 (Figure 21C(c)), leading to an expandable structure, which could more easily absorb and bind water. Therefore, Gel13 exhibited an expandable structure and did not change significantly with an increase in pH.

Novel photo-controlled switching between the water superabsorbent and water-repellent states was demonstrated using TiO_2_-coated, native nanocellulose aerogel networks [109]. In the stable state, these TiO_2_-coated aerogels did not absorb water, and the original absorption and wetting properties slowly recovered upon storage in the dark. In addition to photo-induced absorption and wetting behavior, the TiO_2_-coated nanocellulose aerogels showed also photocatalytic activity, being able to decompose an organic material (methylene blue). Stimuli-responsive hydrogel fibers, with pH-responsive properties, were prepared by electron spinning or coating “smart” polymers on cellulose fibers [42,64]. These fibers showed great potential in the fields of cotton knitwear and biomaterials.

### 3.3. Mechanical-Adaptive Materials

Typical values determined for the elastic or Young’s modulus of plant cellulose fibers have been determined to range between 20 and 30 GPa, but extending up to 138 GPa for highly crystalline cellulose obtained from tensile measurements. And the modulus values obtained from high pressure X-ray diffraction and Roman spectroscopy in a diamond anvil cell were 20 GPa for the bulk modulus and 200–355 and 15 GPa for the crystalline parts and the overall elastic (Young’s) modulus, respectively [152]. Therefore, cellulose nanofibers (also referred to as nanocrystals, nanowhiskers) have been used to reinforce numerous polymer matrices. The significant reinforcement observed for polymer/cellulose whisker nanocomposites can be attributed to the formation of rigid whisker networks in which stress transfer is facilitated by hydrogen bonding between the whiskers. Owing to their strongly interacting surface hydroxyl groups, cellulose nanofibers have a strong tendency for self-association [153]. The mechanical properties of polymer matrices between “on” and “off” states of hydrogen bonding of cellulose nanofibers show great differences. Thus, cellulose nanofibers have been intensively used to fabricate mechanical-adaptive polymer composites.

Capadona *et al.* prepared a cellulose nanofiber network that incorporated EO-EPI and PVAc matrices that were inspired by sea cucumber dermis [124]. The tensile storage moduli (*E*′) of dry sEO-EPI/whisker nanocomposites extracted from the dynamic mechanical analyzes traces for a temperature at 25 °C, in the rubbery regime far above *T*_g_, increased with the whisker content from ~3.7 MPa (neat polymer) to ~800 MPa (19% v/v whisker), and the dry *E*′ was much higher than the swollen *E*′. The observed reinforcement suggested the formation of a percolating nanofiber network in which stress transfer was facilitated by hydrogen–hydrogen bonding between the whiskers. In the swollen state, cellulose whiskers were homogeneously dispersed in the polymer matrix and the hydrogen–hydrogen bonding between cellulose whiskers did not exist because the introduction of water displayed as a competitive hydrogen-bonding agent. Consistent with the proposed mechanism, the mechanical switching was fully reversible: The materials adapted their original stiffness upon drying. The nanocomposites demonstrated significant swelling in both deionized water and artificial cerebrospinal fluid. The solvent uptake increased with whisker content and temperature increase, and lowered the *T*_g_ below the physiological temperature (19 to 23 °C), and reduced *E*′ dramatically. The similar results of PVAc/whisker and PBMA/whiskers nanocomposites were demonstrated in references [125,126] in detail. For SBR or PED/whisker nanocomposites [127], the incorporation of cellulose whiskers into the rubbery polymers increased the *E*′ significantly because a three-dimensional whisker network formed. The reinforcement was primarily on account of the nanofiller–nanofiller interactions, which involved hydrogen bonding. Submersion of these hydrophobic matrix nanocomposites in water resulted in dramatic softening, consistent with disengagement of the cellulose whisker network as a consequence of competitive hydrogen bonding with water. In the same research group’s work published elsewhere [72], xarboxylated CNCs and amine-functionalized CNCs were used as nanofillers to fabricate mechanically adaptive pH-responsive nanocomposites. Carboxylated CNCs filled PVAc exhibited an increase in modulus at low pH, while amine-functionalized CNCs showed the opposite behavior. The neutral or little-charged CNCs showed better mechanical reinforcement than their highly charged counterparts.

**Figure 22:**
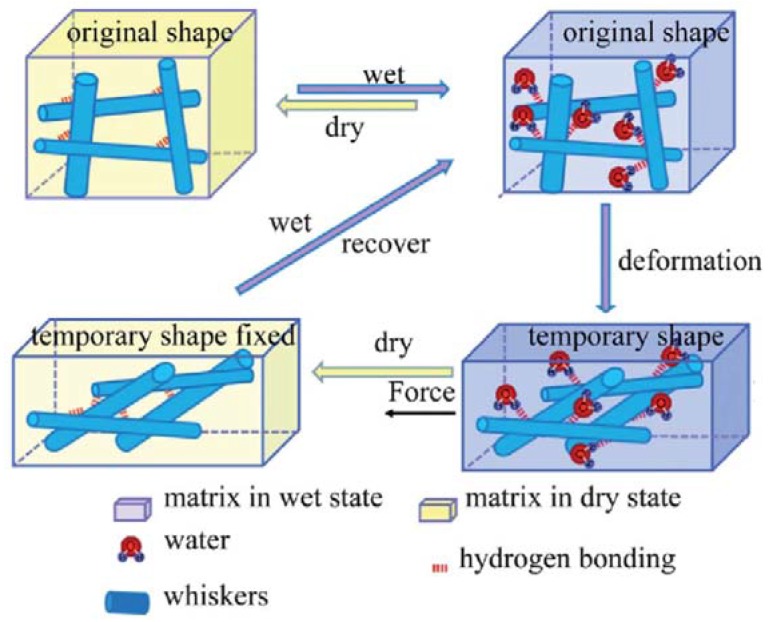
Proposed rapidly switchable water-sensitive shape-memory mechanism for the cellulose nanowhiskers/PUs comprising a cellulose nanowhiskers percolation network in an elastomeric matrix. Reprinted with permission from [117]. Copyright 2012 Royal Society of Chemistry.

Shape-memory materials are mechanical-adaptive materials, which have the capability of changing their shape upon an external stimulus. The movement occurring during recovery is predefined as it reverses the mechanical deformation, which leads to the temporary shape and can be used for self-deploying sun-sails or antennae, morphing wing structures, heat-shrinkable packaging materials, or wrinkle-free fabrics [115]. Many reviews have been published focusing on shape-memory PU materials [113,114,115,116]. Thermal-responsive properties have been described in these reviews, and herein we focus on the shape-memory materials prepared by using cellulose nanofibers as reinforcement and stimulus triggers. Cellulose nanowhiskers (CNW) play the same role as demonstrated above in the thermal sensitive shape-memory CNW/PUs [117,119,120,121,123]. The mechanical properties were improved due to the nanofiller–nanofiller interaction in the polymer and the interaction can be relieved by introduction of water which acted as a competitor of hydrogen bonds. The mechanism of the shape-memory process is proposed in Figure 22 [117].

The combination of a percolation network of cellulose whiskers and an elastomer matrix is the microstructural prerequisite for the rapidly switchable water-sensitive shape-memory effect in these CNW/PUs. Initially (original shape), wetting can soften the CNW/PUs through water molecules attacking the hydrogen bonds between the nanowhiskers. This allows the easy transformation into a temporary shape and the subsequent drying leads to the shape fixation through the formation of a hydrogen-bonded three-dimensional network of individualized whiskers after removal of water molecules. In the recovery procedure, the wetting as external stimulus leads to decoupling of the network of whiskers and triggers the spontaneous shape recovery of the programmed samples. The microstructural characteristics (whisker-network/elastomer) of CNW/PUs and the corresponding shape-memory programming promote the rapidly switchable shape-memory effect. Other work indicated that CNW in the PUs could increase the degree of crystallinity and crystallization rate of reverse phase in the nanocomposites and could engender rapid shape fixity ability after a relative short cooling time [118]. Conductive polyanilline-coated cellulose nanofibrils incorporated in PUs could reduce the electric resistance of the PU composite films, which opened the future possibility of triggering the shape-memory response of these CNW/PUs through use of a stimulus other than temperature [122].

### 3.4. Electro Active Materials

Cellulose paper has recently been discovered to be a “smart” material, termed electro-active paper (EAPap), which is electrically activated due to a combination of ion migration and piezoelectric effect [94,98,154]. EAPap has various applications, such as sensors, actuators, biomimetic robots, flying objects, and haptic materials [95,155,156].

The tip displacement of EAPap is related to composition, fabrication process, humidity, electrical frequency, and voltage. The influences of humidity, electrical frequency, and voltage on the piezoelectric effects are much the same for different EAPap actuators. Increase of humidity can improve the displacement because increasing water content resulting in softening the actuator and making anions easier to move. On the other hand, the resonance frequency is decreased as the humidity level increased [68]. The increase of actuation voltage can also enhance the displacement. The influences of contents of chitosan [157,158], sodium alginate [97], Li [159], and polyurethane [68] in the cellulose paper on the piezoelectric effects were studied. The displacement was increased with the chitosan and sodium alginate content increase due to the increase of number of free ions per unit area, which increased the repelling forces between the anions. Chitosan and sodium alginate were also promising for the reduction of the humidity sensitivity of the EAPap. Higher LiCl content led to higher initial displacement output, but also resulted in a fast decrease with time variation, and lower LiCl content had smaller initial displacement output and slow decreasing rate of the displacement. Interpenetrated PU network was formed in the cellulose actuator and improved the Young’s modulus. However, in high humidity levels (such as 90% RH (relative humidity)) the maximum bending displacement of cellulose/PU EAPap was lower than that of cellulose EAPap, indicating that the cellulose/PU EAPap actuator was more suitable to be used in low humidity. Mahadeva *et al.*, prepared an EAPap actuator based on cellulose polypyrrole-ionic liquid nanocomposite and compared the electromechanical properties with EAPap actuators made by pristine cellulose and cellulose activated by 1-butyl-3-methylimidazolium tetra fluoroborate (BMIBF_4_) [155]. EAPap actuators based on cellulose and BMIBF_4_ activated cellulose showed poor performances and durability. However, after polypyrrole modification, the actuator showed great performance, with nearly 100% improvement of the actuator performance compared to that of pristine cellulose-based EAPap actuator. The introduction of fullerenol into electrospun CA resulted in a substantial increase in crystallinity and mechanical strength, and labile bonding of polymer chains to the fullerenol surface resulted in the formation of novel crystalline structures. Actuation results showed more than 3-fold increase in the tip displacement, even at minute concentrations of fullerenol [99]. Yun *et al.* demonstrated that EAPap actuators based on regenerated cellulose showed three resonance peaks at 0.1, 10, and 40 Hz and the bending displacement of the unimorph EAPap was strongly dependent on the applied voltage, with almost no bending displacement when the applied voltage was lower than 14 V [156]. The authors also illustrated the geometrical effect on the bending performance. A quadratic increase of the resonance peak and a nonlinear decrease in its bending displacement were observed for the shorter unimorph EAPap actuators.

**Figure 23:**
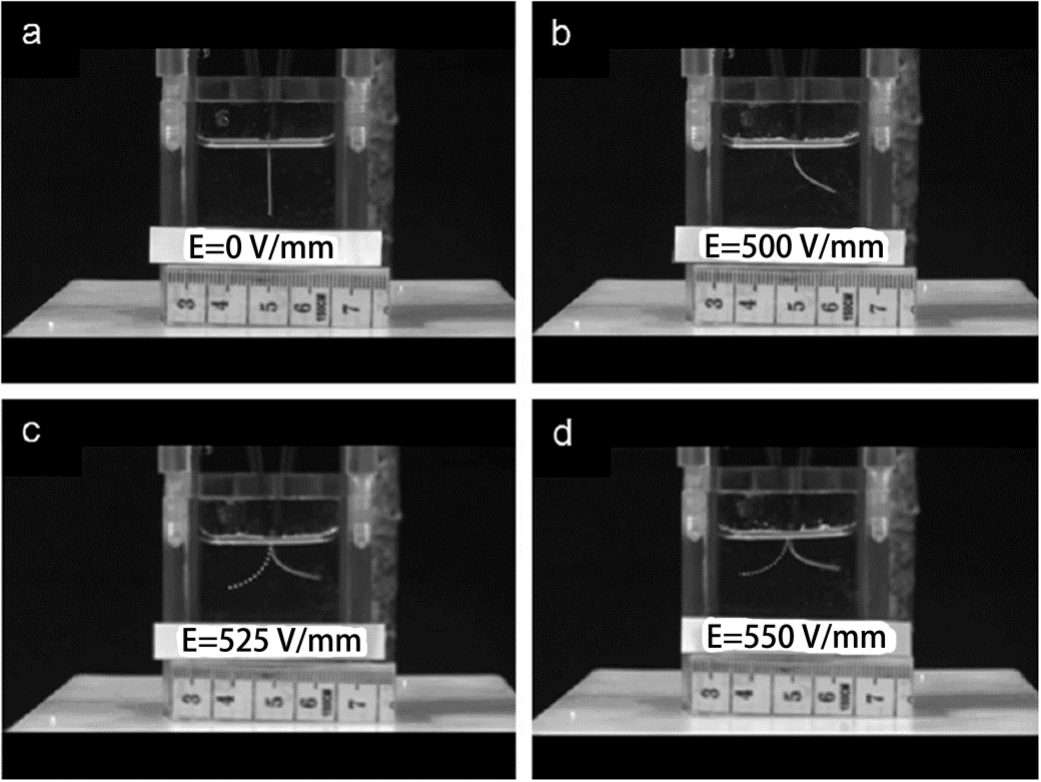
Deflection and back–forth swing images at 303 K under various applied voltages of the gel: (**a**) E = 0 V/mm; (**b**) E = 500 V/mm; (**c**) E = 525 V/mm; and (**d**) E = 550 V/mm. Note: The polarity of the electrode on the left and right hand sides are always GND and positive, respectively. Size of the gel sample: 16.5 mm of length, 1 mm of thickness, 3 mm of width, and 0.0309 g of weight. Reprinted with permission from [100]. Copyright 2012 Elsevier.

The actuation principle of cellulose/sodium alginate EAPap was reported by Kim *et al.* [97]. Sodium alginate and cellulose chains were supposed to be arranged alongside each other because of the long sodium alginate chains. As sodium alginate is an anionic polymer, there are many negative charges (fixed ions) in the molecular chain. Under the condition of low dc voltage, these negative charges could barely move to positive electrode. Conversely, the sodium ions were approximately free and therefore able to move to the negative electrode in the presence of dc voltage. As the sodium ions migrated to the negative electrode, the repelling force between the sodium ions allowed the film to bend in the direction of the positive electrode. The electromechanical properties of the physically crosslinked cellulosic gel were studied elsewhere [100]. The electric field strength induced the internal dipole moment at a relatively low temperature, and the storage modulus enhanced. However, at a relatively high temperature above 313 K, the premature transition temperature and the decreases in the storage moduli, as well as the relative dielectric permittivity, were observed. The deflection experiment showed bending towards the positive side or the anode side under electric field strength above 100 V/mm. The actuation was due to the ionic and electronic polarization via the BMIM^+^ cation and the cellulosic hydroxyl group, respectively. In addition, between 525 and 550 kV/mm, the back and forth swinging was observed due to the competition between the anion and cation movements within the gel (Figure 23).

### 3.5. Sensors

“Smart” materials based on cellulose have vast applications in the sensing field as a result of their behavior changes in response stimulus. Simple optical pH sensors were fabricated by immobilizing pH indicators, such as Methyl Red and phenolphthalein, on cellulose materials [103,160], which showed different colors in response to pH changes. Sensors made by embedding pH indicators in cellulose esters were also used as dissolved ammonia sensors [91], and the sensing properties were resistant to protons in sodium phosphate buffer solution from pH 5 to 8. Conductive materials based on cellulose were prepared as humidity and temperature sensors [92,137]. Series of chemosensors were grafted on cellulose fibers used as cyanide ions sensors in aqueous solution [79]. Immersion of these functionalized textiles in an aqueous solution of cyanide induced a color change linearly correlation with cyanide ion concentration down to 0.01–0.07 μM. Copper and mercury ion sensors were produced by an immobilizing chemosensing agent on cellulose film or fiber by electrospinning [101,102]. The offered chemosensors allowed determination of copper ions in the concentration range of 10^−12^–10^−5^ M and of mercury ions in the large linear working range between 10^−10^–20^−4^ M. Regmi *et al.* prepared an organic vapor-sensitive composite film comprising CA and a representative compound (1-*n*-butyl-2,3-dimethylimidazolium hexafluorophosphate) [86]. The vapor-sensing characteristics of the film were investigated using a quartz crystal microbalance (QCM) transducer. The ratio of change in resonance frequency (Δ*f*) to change in motional resistance (Δ*R*) was a concentration-independent quantity proportional to molecular weight of the absorbed chemical species. Poly(9,9-dioctylfluorene) encapsulated in an amphiphilic cellulose nanocarrier significantly enhanced sensitivity with 50-fold higher quenching efficiency of nitroaromatic explosives in aqueous solutions than in organic solvents [138].

**Figure 24:**
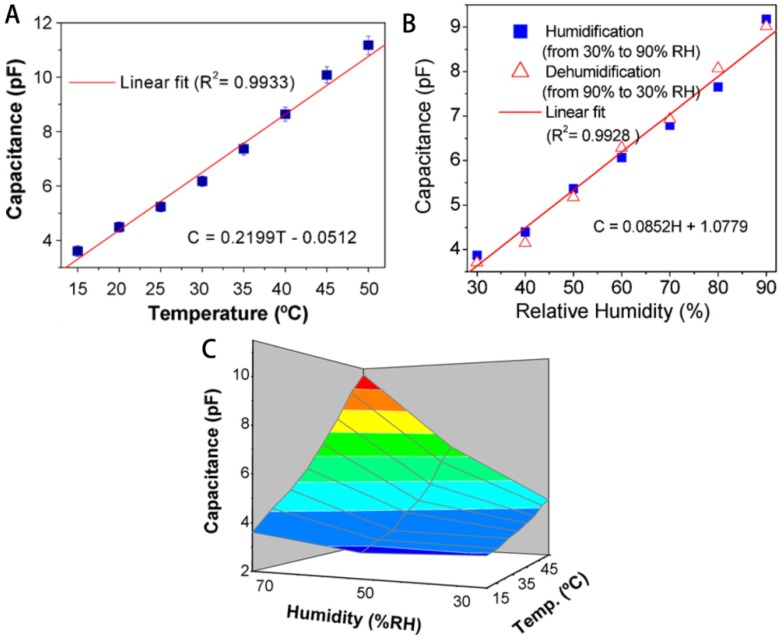
(**A**) Temperature-sensing characteristics at 70% RH; (**B**) Humidity sensing characteristics of cellulose-PPy nanocomposite; (**C**) Capacitance of cellulose-PPy nanocomposite sensor as a function of temperature and humidity. Reprinted with permission from [92]. Copyright 2010 Elsevier.

The capacitance of cellulose-polypyrrole sensor was linear correlation with temperature and humidity (Figure 24A,B) and the capacitance increased as the temperature increased at any given humidity level (Figure 24C) [92]. The humidity-sensing property of nanocrystalline lanthanum ferrite/polymer quaternary acrylic resin was similar to that of cellulose-polypyrrole sensor and the EC coating did not influence the humidity-sensing property but significantly improved the water-resistant property.

### 3.6. Other Applications

Cellulose membranes incorporated or grafted with temperature- and/or pH-responsive polymers can adjust the pore size distribution due to the swelling and shrinking of polymers [63,150]. They were therefore used as anti-fouling membranes in the water-treatment industry and separation industry [74,76]. Smart membrane filters were made by attaching biosensors to temperature-responsive HPC-*g*-EC films for microbial sensing in the water-treatment industry to detect microbial fouling of the membranes [78]. The *N*-vinylformamide-grafted HPC polymers showed good interaction with sodium dodecyl sulfonate and can be used in the water treatment industry for removing surfactants. EC-*g*-Fe_3_O_4_ nanoparticles were found to be interfacially active and magnetically responsive at the oil/water interface and allowed rapid separation of water droplets from emulsions by an external magnetic field [81]. The interfacial activity of EC-*g*-Fe_3_O_4_ nanoparticles allowed them to effectively attach to water droplets in emulsions, while strong magnetic properties of the Fe_3_O_4_ core provided quick and effective separation of the emulsified water droplets from the multiphase systems by magnetic separation.

**Figure 25:**
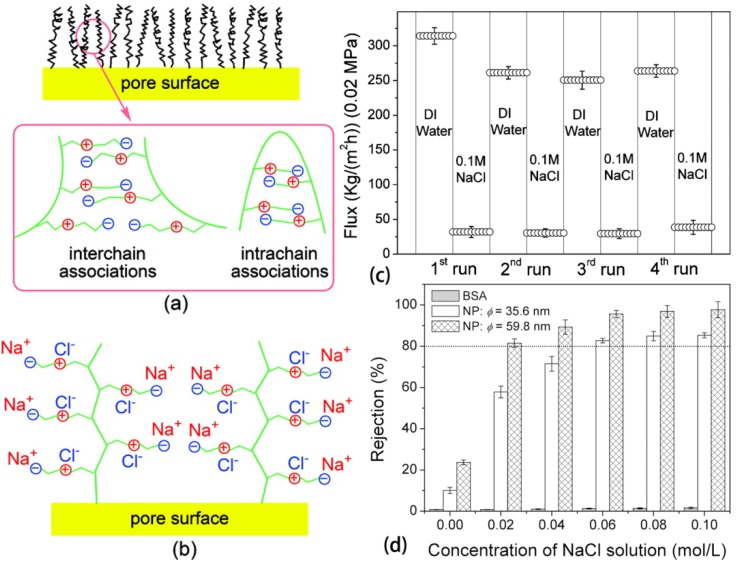
Various characteristic conformational states of PSBMA polymer chains in (**a**) DI water; and (**b**) NaCl solution; (**c**) Reversible electrolyte-responsive behavior of RC-g-PSBMA; (**d**) Dependence of rejection rates of BSA and NPs upon the concentration of NaCl solutions. Reprinted with permission from [161]. Copyright 2009 American Chemical Society.

A previous study showed that pH-responsive hydrogels prepared by grafting crosslinked polyacrylamide onto CMC enabled them to absorb large quantities of solvents, mainly water, in addition to small solutes, while excluding macromolecules such as proteins, and subsequently concentrating them as [37]. An electrolyte-responsive regenerated cellulose membrane grafted with zwitterionic PSBMA could adjust its pore size upon different NaCl concentrations [161]. In water, inter- and/or intra-chain associations would take place because of the electrostatic attractions between the cations and anions, resulting in a collapsed or contract conformation of the PSBMA chains on the pore surface (Figure 25a). After the addition of NaCl, the small Na^+^ and Cl^−^ ions could penetrate to the collapsed PSBMA chains and disrupt the interactions of ammonium and sulfonate groups, leading to an extended conformation of the PSBMA polymer chains, which reduced the effective pore size of the membrane to flow (Figure 25b). Thus, the water flux showed an electrolyte-responsive property (Figure 25c). The membrane allowed BSA to pass through regardless of concentrations of NaCl in the solutions, but for polystyrene nanoparticles (NPs) as the impurity, the rejection rates increased remarkably with the increase of concentrations of NaCl in the solutions (Figure 25d). The membranes with such properties will have a great potential for protein purification and other separation applications. Ekici reported using a CMC/PNIPAm interpenetrated hydrogel for protein purification by adsorption and desorption under suitable temperature and pH conditions [30]. Cellulose matrix incorporated with magnetic nanoparticles showed similar displacement under magnetic fields as EAPap under electronic fields, which has potential applications for microwave desorption and enzyme immobilization [112].

Crosslinked HPC hydrogel membranes with reduced high-temperature diffusivity were applied as flavor-barrier membranes [162]. The thermotropic material comprising of HPMC, NaCl, and water showed temperature and radiation-induced transmittance changes, thus it could be used as an energy efficient window [163]. Cellulose grafted with alkenyloxy substituted cinnamoyl chloride had the potential as LCD materials with photosensitive aligning property [164]. CMC was applied to manufacture temperature-responsive thickeners, which were of great interest as they provide technological solutions for complex fluids that require improved rheological properties above a given temperature [39].

## 4. Conclusions and Outlooks

“Smart” materials based on cellulose show intelligent behaviors in response to stimuli in the vicinity, thus enabling them to be applied in many fields. Cellulose and/or cellulose derivatives, such as CMC, HPC, HEC, EC, in different forms, such as CNCs and films/membranes, have been utilized to fabricate “smart” materials by chemical modifications in homogeneous or heterogeneous conditions, or by physical incorporation. Temperature, pH, electricity, light, salt, magnetic force, *etc.* have been adopted as environmental stimuli to design “smart” materials for different applications. Stimuli-responsive materials based on cellulose have great potential applications in drug delivery systems because of their biocompatibility and biodegradability, where temperature, pH, and magnetic responses are usually applied for targeted drug delivery. Cellulose nanocrystals have been used to manufacture mechanical adaptive materials as reinforcement and water-responsive trigger due to the excellent mechanical strength and strength diminishing in the presence of water. Cellulose has also been applied to fabricate EAPap because of its piezoelectric property. The stimuli-responsive properties of “smart” materials based on cellulose allow them to be used as sensors for detecting pH, humidity, ions, and organic vapors, *etc.* These intelligent materials can also be applied in the water-treatment industry, separation industry, and other industries.

Stimuli-responsive polymers have been intensively studied over the last few years, yet the research of, specifically, stimuli-responsive materials based on cellulose still needs to become the focus of more studies, because the excellent properties allow the materials to be applied in many fields, especially in bioapplications. Although excellent designs of “smart” materials based on cellulose have already been applied successfully in various fields, more work still needs to be done to make them more practical.
